# Naked aggression: Personality and portfolio manager performance

**DOI:** 10.1371/journal.pone.0192630

**Published:** 2018-02-12

**Authors:** Thomas Noe, Nir Vulkan

**Affiliations:** 1 Saïd Business School/Balliol College, U. of Oxford, Oxford, United Kingdom; 2 Saïd Business School/Worcester College, U. of Oxford, Oxford, United Kingdom; Technion Israel Institute of Technology, ISRAEL

## Abstract

We provide evidence that a personality trait, aggression, has a first-order effect on group financial decision making. In a laboratory experiment on group portfolio choice, highly aggressive subjects (measured by a standard psychology test) were much more likely to recommend risky investment strategies consistent with their own personal information, regardless of the information received by other group members. Outside of this group context, aggression had no effect on subject behavior. Thus, our aggression measure appears to capture an aggressive disposition, which seeks to dominate group decisions, rather than simply reflect risk attitudes or cognitive biases.

## Introduction

The risk taking by financial institutions can have dramatic social consequences, as evidenced by the 2008 financial crisis. Thus, it is not surprising that researchers have attempted to identify the determinants of risk taking by financial institutions. This research has focused on the compensation, risk preferences, and cognitive biases of institutional investors. Undoubtedly, this literature has produced many powerful insights into the behavior of institutional investment managers. However, it has largely ignored the social dynamics of institutional investment policy. Because institutional investment decisions are almost always made collectively in a group context, it is quite plausible to conjecture that these dynamics have a significant effect on institutional investor risk taking.

In this paper we propose a new explanation for risk taking, rooted in social psychology—the *aggression hypothesis*: in group decision contexts, a personality trait, aggressiveness (a sub-trait of the Big Five trait neuroticism), affects how fund managers make investment decisions and thus can account for excessive risk taking even in the absence of contractual incentives, cognitive biases, or risk-loving preferences. This line of research is novel and distinct from both neoclassical economics and behavioral economics. Neoclassical economics focuses on rational choice. Behavioral economics focuses on how the *cognitive biases* of *individual* agents distort rational choice. We focus instead on how *personality*, in a *social* context, effects group decisions. Personality is largely stable over adult life, can be measured by validated instruments, and has a significant effect on individual and group behavior [1–4].

We provide evidence supporting the aggression hypothesis through a series of laboratory experiments in which seasoned financial professionals make portfolio allocation decisions. In these experiments, we placed subjects in different decision scenarios. In all of these scenarios, or *treatments*, the subjects were confronted with a choice between a safe and risky asset. In all treatments, given the totality of information available to the subjects, the risky asset was a dominated choice for any risk-neutral or risk-averse investor.

In the baseline treatment, the subjects made a recommendation to a simulated investment group. Each subject received a personal rumor, a signal of the returns on the risky asset, and was informed about the rumors received by other members of the group. When a subject’s personal rumor favored investing in the risky asset, the subject faced a choice: On the one hand, recommending the safe asset is the optimal choice based on expected utility maximization. On the other hand, recommending the safe asset favors a group decision inconsistent with the information the subject personally contributes to the decision. The social psychology literature has associated aggression with a desire for dominance in the group-decision domain. Thus, the aggression hypothesis predicts that, in this sort of group context, more aggressive subjects will be more likely to recommend the risky asset. Our experimental evidence confirms this hypothesis. In fact, a one standard deviation increase in aggressiveness approximately doubled the estimated probability of a subject recommending the risky asset.

The conclusion that this evidence favors the aggression hypothesis rests on two assumptions. (i) Measured aggression, the score of subjects on our instrument, actually identifies the personality trait aggression. (ii) The effect of measured aggression on subject behavior does not simply result from correlation between measured aggression and the individual risk preferences or cognitive biases of the subjects. The social psychology literature has largely established the validity of our aggressiveness instrument and thus we take (i) as a maintained hypothesis. (ii) requires further investigation. The alternative to (ii) is the *proxy hypothesis*: measured aggression correlates with the risk preferences or cognitive biases of the subjects and, outside of this channel of influence, is unrelated to subject behavior.

To evaluate the proxy hypothesis, we implemented three control treatments in which group-decision dominance through signal-correlated recommendations was not possible. The investor characteristics identified in the economic and cognitive psychology literature adhere to preferences over asset returns and beliefs about the probabilistic structure of asset returns. As such, these characteristics do not vary with the social context of the investment decision. Hence, if the proxy hypothesis is correct, then the effects of preference and belief characteristics, proxied by measured aggression, should be insensitive to the social context of decision making. Thus, given the strong effect of aggression on behavior in the baseline treatment, if aggression is proxying for these characteristics, aggression should also have a significant effect on subject choice in the control treatments, which differ from the baseline treatment not with respect to the returns offered by menu of assets but rather with respect to social context. We found that in all of these control treatments, aggression had no significant effect on investor behavior.

Overall, these results support the aggression hypothesis and provide no support for the proxy hypothesis. Thus, they suggest that a personality trait, aggression, has a first-order effect on group investment decisions, an effect that is largely independent of risk preferences and cognitive biases of the individuals composing the group. Highly aggressive group members placed so much weight on dominating the decision process, that they recommend risk-taking strategies that appear to be dominated given their individual risk preferences.

This paper is part of a fast-growing albeit small literature on the effect of personality on portfolio-investment behavior. Most economic research on personality traits has focused on the effect of personality traits on “soft skills” and human capital, e.g., investment in parenting and education (See [5] for a comprehensive discussion). The much smaller body of research on the effect of personality traits on portfolio allocations has primarily focused on personality’s effect on the individual portfolio choices of private investors [6–8]. In contrast, this paper focuses on personality’s effects on allocation decisions by professional investment managers. Perhaps the paper most closely related to this topic is Andersson et al. [9]. Andersson et al. performs a experiment on a random sample of the Danish population. The experiment provided subjects with rewards intended to emulate the high-powered compensation schemes frequently used in the investment-management industry. Subjects individually made portfolio allocations that affected their rewards as well as the value of the simulated investment portfolio. Reward maximization favored investing in the risky asset while portfolio value maximization favored the safe asset. They found that the Big 5 personality factors associated with pro-social behavior, specifically agreeableness and extroversion, mitigated, but did not eliminate, subject risk taking. In contrast to Andersson et al., our study was performed on professional money managers rather than a random population sample, and our aim was not to identify the effects of personality on subject behavior in an individual-decision setting, but rather to identify the effects of personality on professional managers in a group-decision setting.

The effects of personality traits identified in this paper have important implications, both for researchers and practitioners. For researchers, they suggest that group investment behavior cannot be adequately modeled without considering the effect of personality on group dynamics. Our research is but a first step in this direction. We only considered the question of the effect of personality on a desire for decision dominance and not, for example, the question of the effect of personality on the ability to attain decision dominance. We conjecture that other personality traits, e.g., extroversion, might be quite relevant for answering this question. Rather than being thought of as the final word on personality and group investment behavior, our research can best be viewed as a guidepost pointing to a very fertile and largely unexplored field of research: the role of personality in shaping group financial-decision making.

The practical and social implications of these results are perhaps even more significant. Risk taking by financial institutions can cause significant harm to the institutions (e.g., the London Whale and J. P. Morgan) and to the overall economy (e.g., 2008 financial crisis). Identifying a novel covariate that has a first-order effect on risk taking, a covariate that can be easily observed and manipulated at fairly low cost, can greatly increase the effectiveness and efficiency of institutional risk management systems. The personality trait aggression appears, given our results, to be just such a variable. Because personality traits, such as aggressiveness, are largely constant over adult life and can be measured by validated instruments, and because aggressiveness appears to have first-order effects on risk-taking behavior, low-cost interventions aimed to control the average aggressiveness of money managers might significantly reduce the risk of the financial system. Thus, our result suggest practical measures such as personality screening and training, that firms and regulators can undertake to significantly mitigate risk taking in the financial sector.

## Results and discussion

### Methods and hypotheses

#### Subject consent and institutional approval

This experiment was conducted in the Saïd Business School, following the rules and regulations of the Oxford Experimental Laboratory OXLAB (http://oxlab.oii.ac.uk/) where ethical review is standardized for conventional socioeconomic experiments such as this one. This means that the treatment of participants was in agreement with the ethical guidelines of the Central University Research Ethics Committee of the University of Oxford (http://www.admin.ox.ac.uk/curec/). Permission was obtained from the Said Business School Departmental Research Ethics Committee, which is reports to the Social Sciences and Humanities Interdivisional Research Ethics Committee, which in turns reports to the Central University Research Ethics Committee. For full details on the structure of the University research ethics committees please see: https://researchsupport.admin.ox.ac.uk/governance/ethics/committees/drecs#collapse1-0. At the time of the experiment, the Chair of the Social Sciences Interdivisional Research Ethics Committee was Professor Colin Mayer.

Specifically, all participants gave their informed consent to participate voluntarily, assuring them that the analysis and publication of experimental data would be without an association to their real identities. The experiment involved no deception of participants. As in other socioeconomic experiments, there was no additional ethical concerns.

Subjects we emailed in advance to ask if they wanted to participate in the experiment, with brief explanation of what is involved. At the beginning of the experiment we explained again what is involved and how the data will be treated and subjects had the opportunity to leave before the experiment started if they had any concerns. The ethics committee accepted that this procedure (subjects turning up to a written invite, and given verbal explanation again at the beginning the experiment) constituted clear and sufficient consent.

#### Design approach

Our experiment was designed to investigate the effect of personality on the investment choices of individuals making decisions in a group context. Thus, we aimed to isolate the effect of personality on investment preferences. This objective requires collecting the individual choices of the group members before interaction with other members, abstracting from the subjects’ beliefs about future market returns or attractive investment choices as well as from the ability of group members to induce the group to implement their preferred choices. Subject to this constraint we aimed to implement the experiment using the principle of ecological design [10]: designing and framing the problem so that it resembles the problems these subjects typically encounter in everyday life, rather than framing the problem as an abstract calculation problem. Thus, we recruited experienced practitioners for the experiment. In addition we provided subjects with the sort of qualitative information they would likely receive when making a real investment choice. Thus, we did not provide specific probability distributions for the signals or “rumors” the subjects received as such parametric information about signals would never be provided to them in work situations. In addition, we made every effort to make the look and feel of the decision problem as real as possible through our choice of language and the user interface. In the baseline treatment, subjects were placed in groups of five and each received her own signal. The five signals received by the group members appeared on the screen of each group member one after the other, giving the impression of information arriving in real time. Subjects then made an initial “recommendation” which could be accompanied with a message to the group members. We tried to avoid subjects believing, based on past work experience, that risk taking is optimal by framing the problem in a mutual-fund context rather than a hedge fund or proprietary trading context. Despite the fact that the monetary rewards offered in the experiment did not favor risk taking, such erroneous perceptions of trade-offs might bias subjects.

At the same time, consistent with our objective of capturing risk-taking preferences of individuals acting in group context rather than the participants’ opinions regarding specific stocks, we provided only the minimal contextual information required to make the problem appear to be a real investment problem. For example, we never specified the index tracked by the tracking portfolio or the industry in which its alternative, the stock, operates. By specifying that the information signals concern the alternative stock investment, we minimized the effect of agent optimism or pessimism regarding overall economic and/or stock market performance. Because we recorded only the recommendation of subjects, made before interaction, our approach also controlled for the contaminating effect of subjects’ ability to influence other subjects.

#### Specific protocols

Experiment 1 was conducted on January 17th 2014 with 52 participants. Experiment 2 was conducted on September 29th, 2016 and had 72 participants. Both experiments used participants from the (2014 and then 2016) Diploma in Financial Strategy course at the Saïd Business School, University of Oxford (http://www.sbs.ox.ac.uk/programmes/degrees/dfs). All participants gave their informed consent to participate voluntarily, assuring them that the analysis and publication of experimental data would be without an association to their real identities.

In Experiment 1, the gender split was 80% male and 20% female, while in Experiment 2 it was 72% male and 28% female. The demographic distribution of subjects was as similar in both experiments. The participants’ ethnic backgrounds were varied, and, on average, participants had just over 11 years of experience making investment decisions in the Experiment 1 and just under 10 years in Experiment 2. Each experiment lasted about 30-45 minutes. The software used was an online experimental platform which the subjects accessed through their web browsers. The software randomly determined whether a subject encountered the individual or the group experimental task first. Similarly the order of the three treatments in the second experiment was also randomized. The software recorded all inputs from the participants.

Each subject was presented with a choice between passively tracking the index or investing in a stock called “stock A.” In some treatments subjects were told that they were part of a group. Subjects received information signal(s) about the returns to stock A. The signals were called “rumors.” After observing the signal(s) subjects made a decision. In the individual experiments the decision was whether to buy stock A or buy the index. In the group experiments, a subject’s decision was whether to recommend buying stock A or recommend buying the index. In some group experiments, subjects could also send messages supporting their recommendations. In order to simplify the discussion of our results, we will refer to both the choice of buying stock, in the individual experiment, and the choice of recommending the stock, in the group experiment, as choosing to “invest.” Similarly, we will refer to both the choice of the index, in the individual experiment, and recommending the index, in the group experiment, as choosing to “track.”

After a few practice rounds, subjects submitted one decision. The order in which the treatments were performed on subjects was randomized by the software. After completing the investment decision problems, subjects completed the standard Hold and Laury risk assessment task and then answered questions from a survey instrument which encompassed the Big Five, the Rotter Scale (Locus of Control), and the Buss and Perry aggression questionnaire, our instrument for measuring aggressiveness. In addition, they provided demographic information.

At the end of the experiment, four subjects were selected at random and were paid depending on their choices and performance: Consistent with the instructions, they received £20 if they chose to track the index. If they chose to invest in the new stock their pay was determined by a random draw to be either £24 or £14. The probabilities of a high and low draw were determined by the objective Bayesian probabilities of a high or low return. The instructions to the subjects and the visual presentation of the interface software is provided in an supplementary files, S2 and S3 Files. Subjects were not misled in any way by our focus on these instances. Misleading subjects is something we are against and in any case would contravene the rules imposed by our laboratory—see http://oxlab.oii.ox.ac.uk/public/oxlabguidelines.pdf for more details. The distribution of realized subject payoffs for the experiment was conditioned on the same instance reported to the subjects. Thus, our description of the game was consistent with the actual process by which rewards were allocated. Similar designs have been used in other economic experiments such as [11].

#### Treatments

In all treatments the following background, i.e., *prior* information, about the return characteristics of the two assets was provided. Subjects were informed that Stock A, henceforth simply called the “stock,” either earned the index return plus 20% or the index return less 30%. The index returned 20% with certainty. Subjects were advised that, absent any information signals, the high and low return for the stock are equally likely. Given this prior information, a risk-averse or risk-neutral expected utility maximizing investor would always prefer tracking.

In addition, the subjects received a signal, and in all but one of the treatments, observed signals received by other subjects or groups of subjects. In some group treatments, the signals were “personalized,” i.e., subjects were informed that each group member would receive a possibly different signal. In other group treatments, the signals were “uniform” within a group, i.e., subjects were told that the group as a whole received the signal. The signal could either be “good” or “bad.” Subjects were informed that when the future return on the stock is low, a bad signal is more likely than a good signal and, when the future return on the stock is high, a good signal is more likely to be received than a bad signal.

As we show in the supplementary file, S1 File, for an objective Bayesian agent, the revision in the prior probability induced by the observed signals depends only on the difference between the number of good and bad signals. In all designs, the number of bad signals exceeded the number of good signals by one. Thus, for an objective Bayesian agent the posterior information provided by the signals would make investing even less attractive in all treatments.

Our baseline treatment was the *group/personalized* treatment. Subjects were advised that they were members of a group of five. Subjects received a personal signal, “good,” and were told that other group members received three bad and one good signal. Each subject made a recommendation for the group decision: track or invest. In addition to sending a recommendation, subjects could also send a message, providing a rationale for the recommendation. In this treatment the decision is a group decision but subjects had personalized information. If subjects take ownership of this information, then an aggressive disposition, which the social psychology literature has associated with a desire for dominance in the decision domain, should make an aggressive subject more likely than a less aggressive subject to recommend investing after receiving a good signal, and thereby increase the likelihood that her information determines the group decision.

We also implemented three control treatments. In the first, *group/uniform* Subjects were advised that all subjects in their group received the same signal and were also informed about signals received by other groups. In this treatment, the subject’s group received a good signal, the other groups received three bad signals and one good signal. Each subject made a recommendation for the group decision: track or invest. Subjects could not send messages defending their recommendation. In the second, *individual/multiple*, subjects were told that there were 10 other analysts, six of whom received bad signals while four received good signals. They were then told that their own signal was good. Each subject made an individual investment decision: track or invest. In the third, *individual/single*, subjects were told that they received a bad signal. No signals from other analysts were reported. Each subject made an individual investment decision: track or invest.

Each of these control treatments was aimed at parsing the effects of group dynamics on the decision process. The group/uniform treatment was essentially identical to the baseline treatment except that the opportunity to establish decision dominance by ensuring the subjects own information determined the group decision was absent. This treatment was designed to control for the pure effect of switching from an individual-decision context to a group-decision context and the change in framing of the decision as a decision to buy versus recommending to buy. A number of economic theorists (e.g., [12]) have postulated dual self models of decision making in which agents’ individual preference functions are context dependent. In our setting, for example, under the dual-self hypothesis, an agent might have risk-averse preferences when making individual portfolio decisions but risk-loving preferences when making committee investment recommendations. Because both the group/single and baseline group/multiple treatments are framed in a group context, under the dual-self hypothesis, in contrast to the aggression hypothesis, we would expect similar subject behavior in these two treatments.

The two individual treatments abstract entirely from the group setting and thus eliminate the effects of group social dynamics. The two individual treatments were designed to provide a sharp test of whether aggression affects subject behavior only through its correlation with risk preferences and cognitive biases. In these treatments, the aggression hypothesis does not predict an effect of aggressiveness on subject behavior. The individual/single treatment also abstracts from cognitive biases resulting from overweighting own signal relative to the signals received by others. The problematic aspect of this treatment is that the constraint that the number of good signals is one less than the number of bad signals requires that the subject receive a bad signal while the subject receives a good signal in the baseline treatment. Although only the difference between the number of good and bad signals affects Bayesian revisions, the absolute number of signals, whether the signal is good or bad, or is identified with the subject, might affect decision making for subjects with behavioral cognitive biases. For this reason, we also implemented the individual/multiple treatment in which subjects received the good signal and observed many signals.

#### Predictions

In all treatments, deviating from the index by investing is a dominated choice under almost any standard specification of preferences. Subjects were informed that absent any “signals” the stock was equally likely to go up or down. Under this assessment, the expected return on the stock is less than the expected return on the index and the return on the stock is riskier. Moreover, in the experiment, the subjects always received more bad signals than good signals, and were told that bad signals are correlated with low stock returns.

In fact, as we show in the supplementary file, S1 File, a risk-neutral or risk-averse objective Bayesian subject will prefer tracking the index to investing in the stock. In fact, for an objective Bayesian, posteriors will only depend on prior beliefs and the difference between the number of good and bad signals, which is the same in all treatments. Thus, from the perspective of an objective Bayesian subject who, absent information, assigns uniform probabilities to events, the treatments are equivalent. Assuming subjects are either risk averse or risk neutral, the testable prediction of the objective Bayesian hypothesis is therefore that subjects make the same investment decision, track, in all treatments.

Experimental researchers have discovered that Bayesian/Nash predictions of subject behavior are difficult to confirm in experimental settings. Nevertheless, frequently, quantile response models based on Bayesian best replies closely fit subject behavior [13]. In quantile response models, subjects play strategies with a probability that is proportional to the payoff from the strategy in the Bayes/Nash equilibrium. The strategy that yields the highest payoff is not played with probability one. However, the odds that the agent will play a given strategy versus another strategy increase in the difference in the payoffs produced by the two strategies. Given that increased risk aversion increases the payoff difference between the two strategies, the quantile response model predicts that, in both treatments, low risk-aversion subjects are more likely to invest in the stock. Again, the group vs. individual context should have no effect on subject behavior. Thus, the testable prediction of the quantile response formulation is that increasing risk aversion increases the likelihood of tracking.

Our alternative hypothesis, the aggression hypothesis, is that personality, and aggression in particular, matters. Aggressive agents engage in less risky decisions in the gains domain, but more risk taking in the domain of losses [14]. Aggressiveness is associated with a strong desire for dominance over others [15]. Moreover, social psychology research such as [16] has documented that aggression has a stronger effect on behavior in group contexts. Thus, the aggression hypothesis predicts that, in group contexts that afford an opportunity for subjects to exert decision dominance though recommendations, i.e., our baseline treatment, aggressiveness will be positively correlated with choosing to invest rather than track.

### Outcomes

#### Summary statistics

The raw data produced by the experiment are provided in the supplementary files S4 and S5 Files. Summary statistics for the independent variables used in the study are presented in Table 1. As one might expect, given that the experiment recruited financial professionals, the mean age of subjects was fairly high (35) and right skewed. Mean experience has the same characteristics. In contrast to these demographic variables, the instruments used to measure personality factors and risk preferences, except for Rotter scale, exhibited little skewness and less L-Kurtosis than a Normal distribution (L-Kurtosis of the normal ≈ 0.1226). This is not surprising given that the design of these instruments was to some extent shaped by a desire for producing “regular distributions.”

**Table 1:** Summary statistics.

	AGG	AGREE	ROTT	NEURO	OPEN	CONS	EXTRA	RISK	EXP	AGE
Mean	26.80	32.90	16.40	20.90	38.20	34.70	28.50	6.22	10.00	36.60
Median	26.00	33.00	20.00	21.00	38.00	35.00	28.00	6.00	8.00	35.00
Std. Dev.	7.65	5.15	12.30	5.45	4.30	4.91	4.92	2.90	7.03	8.52
Mean Dev.	8.67	5.81	13.90	6.25	4.82	5.56	5.56	3.26	7.63	9.68
L-CV	0.16	0.09	0.42	0.15	0.06	0.08	0.10	0.26	0.38	0.13
L-Skewness	0.10	-0.07	0.06	0.06	-0.00	0.01	-0.03	0.04	0.21	0.10
L-Kurtosis	0.09	0.12	-0.06	0.07	0.16	0.12	0.13	0.11	0.17	0.06

Table 2 presents the sample correlations between the variables measured in the experiment. Except for the unsurprising positive correlation between age and experience, none of the correlations exceed 0.50. Risk aversion’s correlation with the personality variables was, in general, very weak. Perhaps surprisingly, the correlation between risk aversion and aggression was positive *ρ* = 0.12, i.e., aggressive subjects were *more* risk averse on average. This pattern of correlation makes it difficult to argue that aggression is a proxy for risk tolerance. Aggression exhibited a strong negative correlation with agreeableness (*ρ* = −0.44), and, as predicted by psychological theory, it exhibited a fairly strong positive correlation with neuroticism (*ρ* = 0.27).

**Table 2:** Correlation matrix.

	AGG	AGREE	ROTT	NEURO	OPEN	CONS	EXTRA	RISK	GENDER	EXP	AGE
AGG	1.00	-0.44	-0.04	0.27	-0.02	-0.20	-0.05	0.11	0.06	-0.09	-0.16
AGREE	–	1.00	0.07	-0.42	0.07	0.21	0.10	-0.08	0.02	0.10	0.06
ROTT	–	–	1.00	-0.13	0.07	0.23	0.02	0.18	-0.04	-0.03	0.13
NEURO	–	–	–	1.00	-0.04	-0.26	-0.25	-0.06	-0.04	0.03	0.06
OPEN	–	–	–	–	1.00	0.08	0.31	0.04	-0.17	0.13	0.20
CONSC	–	–	–	–	–	1.00	0.08	0.09	-0.13	0.15	0.05
EXTRA	–	–	–	–	–	–	1.00	0.16	-0.16	0.02	-0.12
RISK	–	–	–	–	–	–	–	1.00	0.06	0.03	0.14
GENDER	–	–	–	–	–	–	–	–	1.00	0.14	0.16
EXP	–	–	–	–	–	–	–	–	–	1.00	0.67
AGE	–	–	–	–	–	–	–	–	–	–	1.00

The dependent variable in this study is the subjects’ portfolio decision: track or invest. In the subsequent logistic regressions, the decision to track is coded as 0 while the decision to invest is coded as 1. The number of subjects choosing to invest or track is presented in Table 3. Table 3 reveals that subjects in Experiment 2 were more likely to invest than subjects in Experiment 1 and that the variation in proportion of subjects investing is larger between experiments than between treatments.

**Table 3:** Subject portfolio decisions in the experiments.

Experiment 1			Experiment 2		
*Treatment*	*Decision*		T*reatment*	*Decision*	
	# track	# invest		# track	# invest
group/multiple:	35	10	group/multiple:	42	30
individual/single:	32	13	group/single:	40	32
			individual/multiple:	43	29

The correlations between the track/invest decisions within the two experiments is presented in Table 4. The table reveals that the correlation between the decisions in the baseline treatment, group/multiple, and decisions in the other treatments is fairly low, *ρ* ≤ 0.25. In contrast, in Experiment 2, which implemented two control treatments, group/single and individual/multiple, the correlation between decisions in the control treatments was high, almost 0.60. This suggests that the determinants of portfolio choice in the control treatments, which abstract from group dominance effects, are more similar to each other than they are to the determinants of choice in the baseline treatment.

**Table 4:** Correlations between decisions. The table presents the correlation coefficient between decisions in the treatments implemented in the two experiments, Experiment 1 and Experiment 2.

(a) Experiment 1		
	group/multiple	individual/single
group/multiple	1.00	0.25
individual/single	–	1.00

(b) Experiment 2			
	group/multiple	group/single	individual/multiple
group/multiple	1.00	0.17	0.21
group/single	–	1.00	0.58
individual/multiple	–	–	1.00

### Baseline group/multiple treatment

**Fig 1:**
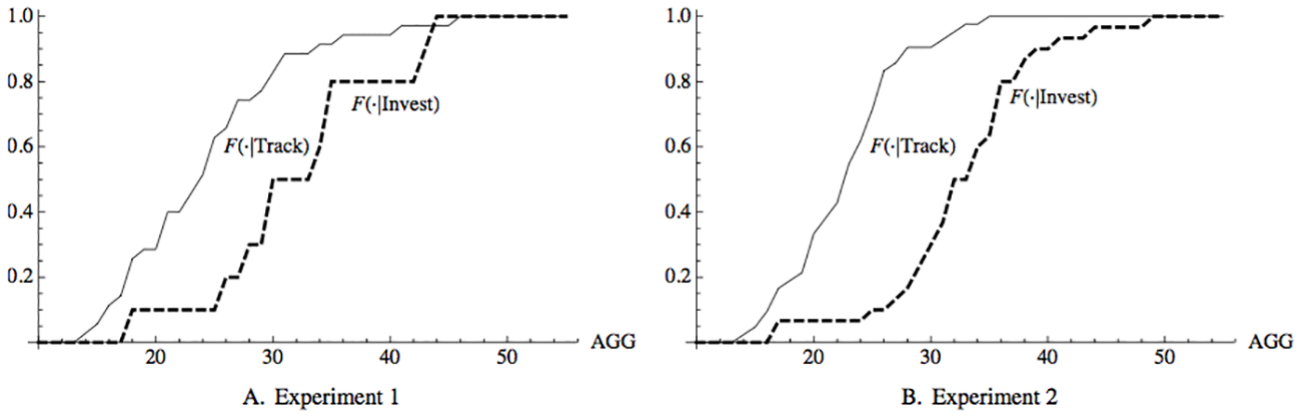
The cumulative distribution of aggression scores conditioned on tracking and investing in Experiments 1 and 2. In each of the panels of the figure, the horizontal axes labeled “AGG” represents subject scores on the aggression instrument. In both panels, the empirical cumulative distributions conditioned on investing (dashed line), *F*(⋅|Invest), and tracking (solid line), *F*(⋅|Track), are depicted.

The key question we are investigating is the effect of aggression on portfolio choice behavior in a group setting. This question is directly addressed by the baseline group/multiple treatment. Fig 1 depicts the empirical cumulative probability distribution of the aggression scores of subjects conditioned on tracking and investing in the two experiments.

As is apparent from Fig 1, in both experiments, measured aggression was much higher for subjects choosing to invest. In fact, the empirical cumulative distribution conditioned on investing in Experiment 2, *F*(⋅|Invest), was stochastically larger, in the sense of first-order stochastic dominance, then the empirical distribution conditioned on tracking. In Experiment 1, it was nearly stochastically larger. Fig 1 provides very direct evidence that measured aggression was strongly positively correlated with investing.

This evidence is confirmed by standard univariate statistical tests in Table 5. The table presents a representative non-parametric test, Mann-Whitney, and a parametric test, a logistic regression. The Mann-Whitney test statistic *U* is used to evaluate the null hypothesis that the median aggression score of tracking subjects is the same as the median aggression score of investing subjects. The test decisively rejects the null hypothesis in both experiments. In the logistic regression, the coefficient for the aggression measure is positive and highly significant.

**Table 5:** Effect of aggression in the baseline treatment. The table presents the results of the non-parametric Mann-Whitney test of the null hypothesis that the median aggression scores of tracking and non-tracking subjects are equal as well as a univariate logistic regression in which the dependent variable is the decision between tracking and investing and the independent variable is the aggression score.

(c) Experiment 1				
			Mann-Whitney Test	
	*μ*-Invest	*μ*-Track	*U*	P-Value
AGG	32.3	24.6	275	0.006
#Obs.	10	35	–	–
	Logistic Regression			
	Estimate	Std. Error	*z*-score	P value
Intecept	-4.835	1.584	-3.053	0.002
AGG	0.127	0.0519	2.448	0.0143
(d) Experiment 2				
			Mann-Whitney Test	
	*μ*-Invest	*μ*-Track	*U*	P-Value
AGG	32.9	23.0	1130.0	1.45 × 10^−8^
#Obs.	30	42	–	–
	Logistic Regression			
	Estimate	Std. Error	*z*-score	P value
Intecept	-8.74784	1.956	-4.471	7.795 × 10^−6^
AGG	0.303	0.069	4.409	0.00001

In order to investigate the possibility of confounding effects, we performed a multivariate logistic analysis, including as controls the other personality variables, the risk-aversion measure, and demographic variables. The results of these regression estimates are provided in Table 6. Once again the coefficient associated with aggression is positive and very significant in both experiments. None of the coefficients associated with the other variables are significant at the conventional 5% level. Both experiments Experiments 1 and 2 implement the baseline treatment. However, the alternative treatment to the baseline treatment is different in the two experiments. Thus, in order to control for the effects of this variation, we have presented estimated separate regressions for the two treatments. However, assuming that experience in other treatments did not affect subject behavior in the baseline treatment, the power of the tests can be greatly increased by combining the treatments without incurring bias. Thus, we also present the multivariate logistic results combining the results from the baseline treatments in E1 and E2 in Table 7. The results of the combined analysis in Table 7 are quite consistent with the results for the individual experiments in Table 6. The only notable difference is that ROTTER, an alternative measure of aggressiveness, is significant in the expected (positive) direction in the combined dataset but not significant in the datasets for either individual experiment. This pattern is consistent with the increased power of the regression using the combined data set. However, the p-value associated with ROTTER in the combined regression is still much higher than the p-value associated with measured aggression, AGG.

**Table 6:** Determinants of investing vs. tracking in the baseline treatment. The table presents the results of a multivariate logistic regression in which the dependent variable is the invest/track decision and the independent variables are personality, risk preference, and demographic information for the subjects.

(a) Experiment 1				
	Estimate	Std. Error	z-Statistic	P-Value
Intercept	-3.720	8.430	-0.441	0.659
AGG	0.166	0.076	2.190	0.028
AGREE	0.025	0.112	0.226	0.821
ROTTER	0.230	0.458	0.503	0.615
NEURO	-0.010	0.089	-0.113	0.910
OPEN	-0.054	0.132	-0.410	0.682
CONS	-0.065	0.107	-0.606	0.545
EXTRA	-0.013	0.096	-0.136	0.892
RISK	-0.061	0.302	-0.203	0.839
GENDER	-0.511	1.440	-0.355	0.723
EXP	-0.025	0.088	-0.278	0.781
AGE	0.062	0.076	0.812	0.417
(b) Experiment 2				
	Estimate	Std. Error	z-Statistic	P-Value
Intercept	-21.000	8.720	-2.410	0.016
AGG	0.352	0.085	4.160	0.000
AGREE	-0.002	0.102	-0.017	0.987
ROTTER	-0.071	0.085	-0.839	0.401
NEURO	-0.007	0.088	-0.083	0.934
OPEN	0.168	0.108	1.560	0.120
CONS	0.203	0.114	1.780	0.076
EXTRA	0.046	0.090	0.504	0.615
RISK	0.015	0.123	0.124	0.901
GENDER	0.498	0.901	0.552	0.581
EXP	0.131	0.084	1.570	0.117
AGE	-0.102	0.080	-1.280	0.199

**Table 7:** Determinants of investing vs. tracking in the baseline treatment combining E1 and E2. The table presents the results of a multivariate logistic regression in which the dependent variable is the invest/track decision and the independent variables are personality, risk preference, and demographic information for the subjects.

	Estimate	Std. Error	z-Statistic	P-Value
Intercept	-11.00	5.130	-2.150	0.032
AGG	0.230	0.047	4.860	0.000
AGREE	0.008	0.060	0.131	0.896
ROTTER	0.048	0.023	2.040	0.042
NEURO	-0.007	0.055	-0.129	0.897
OPEN	0.064	0.071	0.903	0.366
CONS	-0.003	0.059	-0.058	0.954
EXTRA	0.016	0.060	0.261	0.794
RISK	0.012	0.090	0.136	0.892
GENDER	-0.146	0.636	-0.229	0.819
EXP	0.038	0.054	0.706	0.480
AGE	-0.003	0.046	-0.077	0.939

Thus, in the individual experitment baseline treatments, measured aggression, AGG, is the only significant predictor for deviations from the market portfolio. In the combined data set, measured aggression and ROTTER, an alternative proxy for aggression, are the only two significant predictors. Granting that aggression is a significant predictor of deviations from market tracking, naturally raises the question of the magnitude of the aggression effect. To answer this question, we compared the probability of investing of an investor with a mean level of measured aggressiveness, *μ*, with the probability of investing of an investor with measured aggressiveness one standard deviation above the mean, *μ* + *σ*, using the coefficient estimates form the multivariate logistic model (very similar results were obtained using the univariate estimates). Our results show that in both experiments, the magnitude of the aggression effect is quite large. In Experiment 1, an increase of one standard deviation increased the estimated probability of investing from 0.184 to 0.383. In Experiment 2, an increase of one standard deviation increased the estimated probability of investing from 0.372 to 0.851. Thus, in both experiments, a one-standard deviation increase in measured aggression roughly doubled the probability of investing.

In order to further evaluate the aggression/investing relationship we performed robustness tests not reported in the tables. For each variable, in both experiments, we investigated, using both logistic and Mann-Whitney tests, whether, in a univariate setting, the variable predicted investing. We could not reject the null hypothesis of no effect for any of these other variables. Thus, the experimental results in the baseline treatment provide overwhelming support for the hypothesis that in group decisions where group members can vie for decision dominance, aggression has a first-order effect on behavior. An affect that is not captured by standard measures of individual risk preferences or by other personality factors.

#### Control treatments

The baseline treatments show that measured aggression significantly affects investor behavior. The aggression hypothesis asserts that this effect is produced by the channel identified in social psychology, decision dominance in group settings. The most plausible alternative hypothesis consistent with the results in the baseline treatment is the *proxy hypothesis*: Measured aggression is highly correlated with the characteristics of subjects’ preferences or beliefs. These preferences and beliefs have first-order effects on subjects’ portfolio allocations. Measured aggression effects behavior because it acts as proxy for these characteristics. The control treatments aim to evaluate the plausibility of the proxy hypothesis. The characteristics of preferences and beliefs identified in the economic and cognitive psychology literature adhere to preferences over asset returns and beliefs about the probabilistic structure of asset returns. As such, these characteristics do not vary with the social context of the investment decision. Hence, if the proxy hypothesis is correct, then the effects of preference and belief characteristics should be insensitive to the social context of decision making. Thus, given the strong effect of aggression on behavior in the baseline treatment, if aggression is simply proxying for these characteristics, aggression should also have a significant effect on subject choice in the control treatments, which differ from the baseline treatment not with respect to the returns offered by menu of assets but rather with respect to social context.

For example, if measured aggression proxies for risk-loving preferences, then aggressive subjects should be more likely to invest in all of the control treatments. If aggression proxies for subject confidence in the quality of the signal, than in the individual/single treatment, aggressive subjects should be *less* likely to invest, as in this treatment the only signal they observe is unfavorable to investing. If aggression proxies for subject arrogance, overweighting the quality of a subject receives relative to other signals observed, then in the group/single treatment aggressive subjects should be more likely to invest as, in this treatment, the subject’s own group received a signal favoring investment. The effect of measured aggression in control treatments is presented in Table 8. The results in Table 8 reveal that in the control treatments measured aggression had essentially no effect on subject decisions. In the control treatments, the hypothesis that the aggressiveness coefficient, AGG, equals 0 cannot be rejected at even the 10% level of confidence. We also tested for a relation between aggression and investing using non-parametric tests and report the results for the Mann-Whitney test. Again, these tests fail to confirm a significant relation between aggression and investing.

**Table 8:** Aggression and investing in the control treatments. The table presents the results of univariate Logistic regressions in which the decision, to invest is the dependent variable and the independent variable is measured aggression as well as the results of a Mann-Whitney test for the null hypothesis that the median aggression scores of investing and tracking subjects are equal.

Treatment	Logistic regression estimates				Mann-Whitney Test	
	Estimate	Std. Error	*z*-score	P value	*U*	P value
*Group/Single*						
Intecept	-1.605	0.939	-1.708	0.088		
AGG	0.052	0.033	1.527	0.127	742.000	0.245
*Indvidual/Multiple*						
Intecept	-1.110	0.923	-1.200	0.230		
AGG	0.026	0.033	0.805	0.421	657.000	0.696
*Indvidual/Single*						
Intecept	-1.200	1.150	-1.050	0.294		
AGG	0.011	0.041	0.278	0.781	227.000	0.634

In order to further investigate the difference between the determinants of portfolio choice in the baseline and control treatments, we performed a number of robustness tests not reported in the tables. First, we performed a multivariate logistic regression analysis on each of the control treatments. In all of these regressions, the coefficient associated with aggression was insignificant at the 10% level. Other personality and risk-preference factors did exhibit some predictive power in the control treatments. In the group/single and individual/multiple treatments, univariate and multivariate logistic regressions as well as the Mann-Whitney test identified the personality trait openness as a significant predictor of investing. In the individual/single treatment, risk aversion was identified as a significant predictor of tracking by the Mann-Whitney test. Because the context of individual/single treatment is individual portfolio choices by a single investor, the classic context of individual choice portfolio allocation problems, this result provides some assurance that our instrument for measuring risk aversion, the Holt-Laury questionnaire, is valid. However, in the multivariate models for the three control treatments, the Likelihood ratio test does not reject the hypothesis that the likelihood ratio of the multivariate model is the same as the likelihood ratio of a null model that includes only an intercept term. Given the large number of independent variables relative to the sample size in these regressions, this not too surprising.

Nevertheless, it does highlight much stronger predictive power of aggression in the baseline treatment relative to the predictive power of risk and personality measures in the control treatments. In summary, in the control treatments, aggression did not have first-order predictive power for subject behavior either in absolute terms or relative to other covariates. The control treatments differ from the baseline treatment because they abstract from decision dominance. The opportunity to exercise decision dominance is irrelevant to cognitive and economic models of asset allocation, but highly relevant to the expression of an aggressive disposition. Thus, the control treatments provide no support for the proxy hypothesis.

## Conclusion

This paper considered the effect of personality trait, aggressiveness, on professional managers’ portfolio allocations in a group setting. We found that this personality factor had a very significant effect on behavior, approximately doubling the probability of recommending risky investing strategies. The results also suggest that aggression’s effect on behavior is not simply an artifact of aggressiveness being correlated with properties of beliefs and preferences used by investors to make personal portfolio allocations. In short our results point to a hitherto ignored “elephant in the room”— the effect of personality on risk taking in the social context of the finance industry. We investigated whether a *prima facie* important non-cognitive trait of fund managers, one that financial firms routinely screen for in hiring—aggressiveness—effects fund manager behavior. We documented strong, economically significant effects of personality on behavior. Given the externalities generated by risk taking by financial firms, our results suggest that the managerial personality may significantly affect financial stability.

Admittedly, this paper is a first not a last step in parsing the effect of aggression, and personality in general, on the behavior of professional investors. Although we point to the elephant, we do not provide an explanation of how the elephant got into the room. More theoretical research is required to develop a plausible model of how personality is mediated by preferences, information, and incentives, to produce decisions. More empirical research is required to validate the results of this experiment in the field and explore how other personality factors, such as extroversion, affect the ability of aggressive agents to bend group decisions in their preferred direction. Admittedly, this research will be difficult. The link between personality factors and the economic model of choice is even more “awkward” than the link between the economic model and cognitive biases. However, as with elephants, the fact that a factor is awkward does not imply that it is not powerful or that it can be safely ignored.

## Supporting information

S1 File Online appendix. This file provides derivations of the Bayes rational decisions for the subjects under various assumptions about prior beliefs and preferences.
(PDF)

S2 File Instructions. (PDF)

S3 File Questionnaire. (PDF)

S4 File Raw data for Experiment 1. (PDF)

S5 File Raw data for Experiment 2. (PDF)
