# Supplementary material for: Naked aggression: Personality and portfolio manager performance
Source: PLoS One. 2018 Feb 12;13(2):e0192630. doi: 10.1371/journal.pone.0192630 (PMC5809062; doi:10.1371/journal.pone.0192630)
Supplement: S1 File — This file provides derivations of the Bayes rational decisions for the subjects under various assumptions about prior beliefs and preferences. (PDF) [file pone.0192630.s001.pdf]

Online supplement for
Naked Aggression: Personality and portfolio manager performance

Thomas Noe
Saïd Business School/Balliol College, Oxford*

Nir Vulkan
Saïd Business School/Worcester College†

10th August, 2017

A Bayes rational decision problem

A.1 Decision of a risk-neutral Bayesian subject

In this section of the supplement, we consider the Bayes rational solution to the participants' decision problem and belief-based deviations from Bayes rationality that might explain investment choices. We focus on the group experiment because it is central to testing our hypotheses regarding the effect of aggression on portfolio manager behavior. Because the decision problem is very simple, the analysis of the problem produces no surprising results. To facilitate the discussion of these results, we will say that signal quality is symmetric if (a) the signal is equally precise when conditioned on a high or low return on the stock, and (b) all subjects receive signals of equal quality.

Let S represent the proposition that three bad signals and two good signals about the investment have been received. Let H represent the proposition that investing will generate the high payoff of £24; Let L represent the proposition that investing will generate the low payoff of £14. Let q represent the investors prior assessment that the high payoff will be realized. Let γ represent the probability that the signal is good given that the high payoff will be realized; Let β represent the probability that the signal is bad given that the low payoff will be realized. The only information given to the subjects regarding q, γ, and β was that the prior probability that the investment would yield a high payoff equaled $1/2$ and that good signals are more likely when the investment will yield a high payoff and bad signals are more likely when the investment yields a low payoff, i.e.,

$$q = {}^1\!/\!_2, \quad {}^1\!/\!_2 \le \gamma \le 1, \quad {}^1\!/\!_2 \le \beta \le 1. \tag{A-1}$$

*Park End Street, Oxford, OX11HP, UK. email: thomas.noe@sbs.ox.ac.uk

†Park End Street, Oxford, OX11HP, UK. email: nir.vulkan@sbs.ox.ac.uk

First consider the optimal decision absent information from the signals. The decision to track produces a riskless cash flow while the decision to invest produces a stochastic cash flow. Thus, if the expected cash flow from investing is less than the expected cash flow from tracking, risk averse investors will prefer tracking. Given that $q = 1/2$ the expected payoff from investing is less than the expected payoff from tracking. Thus, based on prior information, all risk neutral or risk averse investors will prefer to track. In the individual decision experiment, subjects received only one signal, a negative signal, thus their posterior assessment should be even lower than their prior assessment and hence, a fortiori, all risk averse or risk neutral subjects should prefer tracking in the individual decision experiment.

Now consider the optimal decision conditioned on the signals in the group decision experiment. Our benchmark is the optimal decision of a Bayesian risk-neutral investor. The utility of a risk-neutral investor if she invests equals the expected payoff from the investment conditioned on S, while The utility of a risk-neutral investor if she tracks is £20. Thus, investing will produce a weakly higher expected payoff than tracking if and only if

$$24\mathbb{P}[H|S] + 14\mathbb{P}[L|S] \geq 20. \tag{A-2}$$

Because there are only two possible payoffs from investing, high and low, and the payoff from tracking is riskless, the optimality of investing versus tracking is entirely determined by the relative odds of a high payoff versus a low payoff. Investing is an optimal strategy if and only if

$$\text{odds} = \frac{\mathbb{P}[H|S]}{\mathbb{P}[L|S]} = \frac{\mathbb{P}[H\&S]}{\mathbb{P}[L\&S]} \geq \frac{3}{2}. \tag{A-3}$$

where the equality

$$\frac{\mathbb{P}[H|S]}{\mathbb{P}[L|S]} = \frac{\mathbb{P}[H\&S]}{\mathbb{P}[L\&S]}$$

follows from Bayes rule. Thus, a necessary condition for a risk averse investor to invest rather than track is that the relative odds of a high payoff versus a low payoff at least equal 3 : 2.

Now consider the decision problem of a risk-neutral Bayesian subject whose beliefs are based only on the information provided in the experiment's instructions. The subjects' information is given by equation (A-1). Since this subject has no reason to believe that any value of the parameters γ, and β) within the specified ranges is more likely than any other, she will assign a uniform probability distribution over the specified ranges to these parameters. Thus, subject beliefs about these parameters will be represented by a uniform distribution over the range of specified values, i.e.,

$$\tilde{\gamma} \stackrel{\text{dist.}}{=} \text{Unif}[1/2, 1], \quad \tilde{\beta} \stackrel{\text{dist.}}{=} \text{Unif}[1/2, 1].$$

Because, no information has been provided regarding the relation between these parameters, an objective Bayesian subjects beliefs about the realization of any one parameter will be independent of her beliefs about the others.[1] Thus, the joint probabilities of H and S, to an objective Bayesian subject who relies only on the information provided by the instruction sheet, are

[1]See Jaynes (2003) for a discussion why these probability assignments optimally mirror the subject's information.

represented by $H\&S$ and L and S, represented by $L\&S$, and are given as follows:

$$\mathbb{P}[H\&S] = \mathbb{E}\left[1/2 \binom{5}{2} \tilde{\gamma}^2 (1-\tilde{\gamma})^3\right] = \frac{11}{384}, \tag{A-4}$$

$$\mathbb{P}[L\&S] = \mathbb{E}\left[1/2 \binom{5}{2} \tilde{\beta}^3 (1-\tilde{\beta})^2\right] = \frac{7}{128}. \tag{A-5}$$

Therefore, combining equations (A-4) and (A-5) we find that an objective Bayesian subject would assign odds of

$$\text{odds} = \frac{\mathbb{P}[H\&S]}{\mathbb{P}[L\&S]} = \frac{11}{21}.$$

Since the subject's odds are 11 : 21 and the required odds for investing are 3 : 2, a risk-neutral objective Bayesian subject would decisively reject investment in favor of tracking. A risk averse objective Bayesian would require even better odds, and thus would always reject. Thus, for a weakly risk averse objective Bayesian subject investing in the index is the optimal decision.

A.2 Asymmetric signal quality based the return realization

Rationalizing a subject's decision to invest in the stock requires relaxing at least one of the two symmetry conditions. We first consider the effect of relaxing the assumption that the precision of the good and signals is the same. Consider a subject who assigns the same signal quality to her signals and the other subject's signals but has arbitrary beliefs the quality of the good and bad signals measured by β and γ. The odds for this subject are given by

$$\text{odds} = \frac{1/2\binom{5}{2}\gamma^2(1-\gamma)^3}{1/2\binom{5}{2}\beta^3(1-\beta)^2} = \frac{q\gamma^2(1-\gamma)^3}{(1-q)(1-\beta)^2\beta^3} \quad \gamma \in [0.50,1],\ \beta \in [0.50,1],\ q \in [0,1].$$

In order the subject to invest, it must be the case that the subject's beliefs generate odds at least equal to 3 : 2. The set of parameters supporting investing and tracking under these assumptions are illustrated in Figure 1.

From Figure 1 we see that any subject belief under which $\gamma = \beta$, i.e., any belief under which the estimated precision of the signal is independent of the future realized cash flow, will lead the subject to track rather than invest. Investing is only supported by the belief that the signal is much more precise when the realized cash flow is low. In this case, if the realized cash flow is low, the subject expects almost all the signals to be bad while if the cash flow is high, the signals will be more or less random. Since, in the group experiment, the number of good signals is close to the number of bad signals, and thus fairly "random," such a subject would infer from the high degree of variation in the signals that the return will be high based on her belief that signal variance is much higher when the realized payoff is high. As Figure 1 shows, such posterior assessments are only supported by rather extreme beliefs regarding the asymmetry of signal quality between good and bad signals. Moreover, note that the graph in Figure 1 only depicts deviations from tracking under the assumption of subject risk neutrality. Because deviation also increases risk, risk aversion will further reduce the region over which the subject will choose to deviate from the tracking. Thus, subjects with a moderate degree of risk aversion will only deviate to investing under even more extreme beliefs about signal quality asymmetry. We doubt that this sort of asymmetric assessment of signal quality played

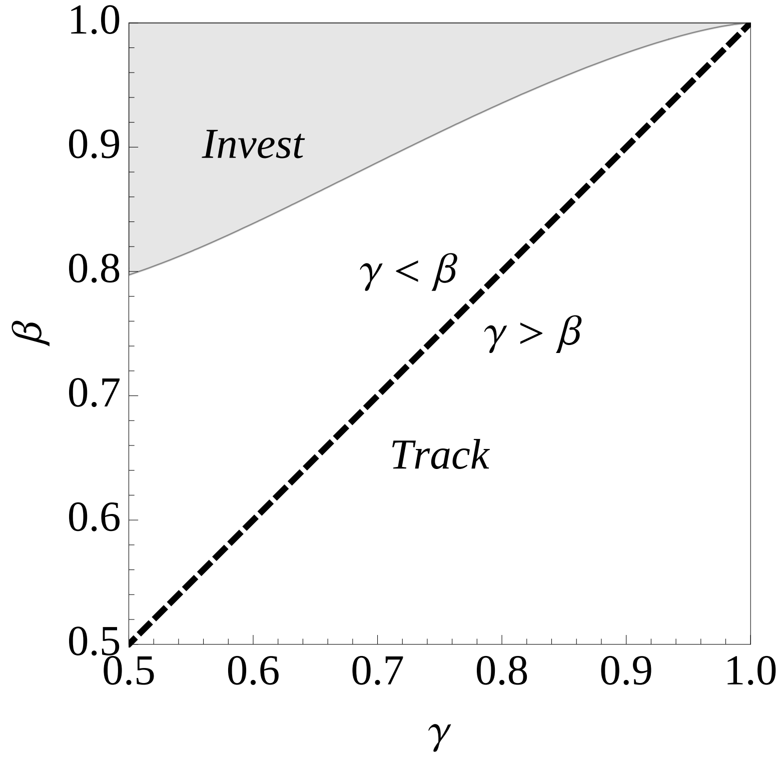

Figure 1: *Asymmetric beliefs about signal quality.* The horizontal axis represents γ, the probability that an individual signal is good given that the payoff from investing is high. The vertical axis represents β, the probability that an individual signal is bad given that the payoff from investing is low. The Investors prior probability equals $1/2$.

an important role in subject decision making for three reasons: First, the degree of asymmetry required to support investing in the stock is extreme. Second, this sort of asymmetry is not motivated by the experiment's instructions. Third, the line of reasoning connecting this sort of signal asymmetry to posterior assessments is quite complex.

A.3 Overweighting of own signal

Now consider the effect of relaxing the assumption that the subject views her own signal as no better or worse than the signals received by the other subjects. In particular, consider a subject who believes that his own signal is more informative than the signals received by other agents, i.e., the subject "overweights" his own signal. To abstract from the issue of asymmetric quality of good and bad signals suppose that under the subject's subjective beliefs are that good and bad signals have the same quality, i.e., $\beta = \gamma$. Assume that the subject assess is own signal quality as equal to o and the quality of the other subjects signal quality is equal to $\beta = k\,1/2 + (1-k)\,o$, $k \in [0,1]$. Thus, overweighting equal to 1 corresponds to the belief that the other subjects have completely worthless signals, $\beta = 1/2$, while overweighting of 0 corresponds to the belief that the quality of other subject signals is the same as the subject own signal. When making the decision, an overweighting subject will consider his own signal—good, and the four signals received by the other agents in the group—three of which are bad and one of which is good.

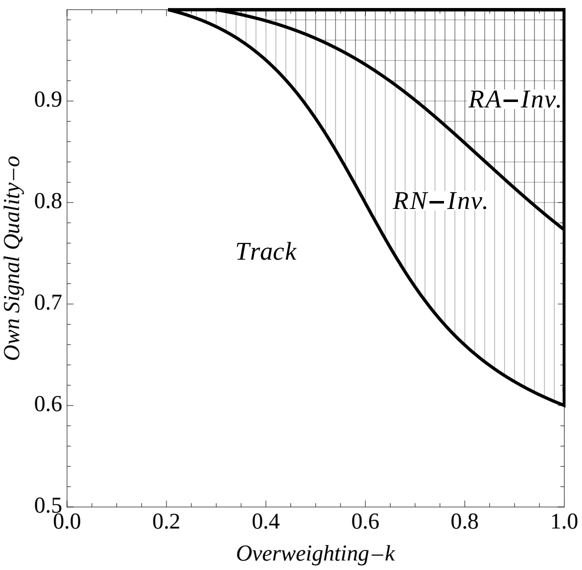

Figure 2: *The effect of overweighting of own signal.* In the figure, the horizontal axes represents the degree to which a subject attributes excess precision to her own signal relative to the signals of the other subjects. The vertical axis represents the subject attribution of precision to her own signal. The vertically hatched region labeled "RN-Inv" represents the set of parameter value under which the subject will invest if the subject is risk neutral. The horizontally hatched region labeled "RA-Inv" represents the set of parameters under which the subject will invest if the subject is risk averse with CPRA utility (coefficient of risk aversion equals 3).

Thus, the subject's odds ratio equals

$$\frac{o\beta\,(1-\beta)^3}{(1-o)\,(1-\beta)\,\beta^3} = \frac{o\left(1-\frac{k}{2}-(1-k)\,o\right)^2}{(1-o)\,\left(\frac{k}{2}+(1-k)\,o\right)^2}$$

As shown above for a risk neutral investor to invest rather than track the market, the odds ratio must at least equal 3:2. If the investor exhibits a high, but reasonable given experimental evidence, level of risk aversion, say the investor has constant proportional risk aversion with a coefficient of 3, the probability that investing will produce the high return must at least equal 0.773 implying an odds ratio of approximately 17:5. Figure A.3 depicts levels of overweighting k and perceived own signal quality o under which the investor will invest and track. From the Figure we see that overweighting as well as a significant degree of confidence in own signal and that the degree of overweighting and confidence required increases with subject risk aversion.

A.4 Economic significance of losses from investing rather than tracking

Finally, suppose that the subject has Bayesian objective posterior beliefs. In this case, tracking is always optimal. However, a natural question to ask is whether the losses from investing relative to tracking are economically significant. To address this question we plot the percentage loss from investing relative to tracking of certainty equivalent wealth for a range of possible risk aversion levels. We assume that the investor's utility is specified by a constant proportional risk aversion (CPRA) utility of wealth function with risk aversion coefficient a. To check the

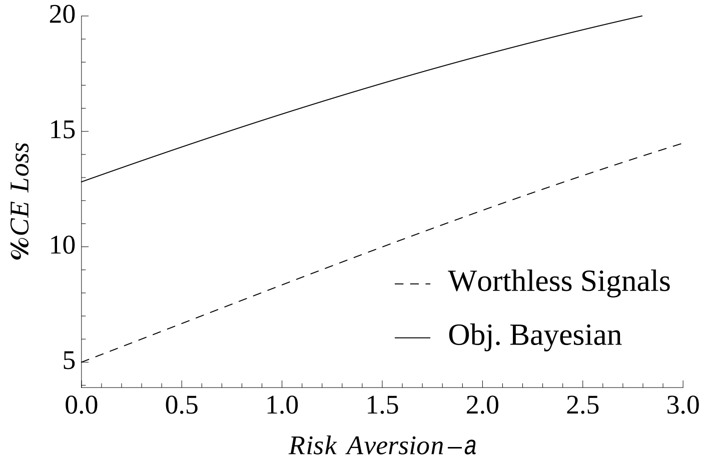

Figure 3: *Certainty equivalent loss from tracking*. In the figure the horizontal axis represent the CPRA risk aversion coefficient of the agent. The vertical axis represents the certainty equivalent loss from investing relative to tracking. The "Objective Bayesian" line represents the loss to an agent who forms posterior assessment based on the updating procedure outlined in Section A.1; The "Worthless signals" line represents the loss to an agent who forms posterior assessment based on the belief that both good and bad signals are uninformative.

robustness of our conclusions, we also graph the percentage certainty equivalent loss assuming that the investor believes that the signals are worthless, i.e., $\beta = \gamma = 1/2$. The case were the signals are worthless represents a lower bound on the losses to subjects who believe that the signals are conditionally independent and signal quality is symmetric. As one can see from Figure A.4, for an objective Bayesian subject, the loss from investing relative to tracking in percentage terms, ranges from 12% to 20%. Even under the assumption that the signals are worthless, loss from investing is at least 5%. Thus, the decision to invest rather than track will have a significant effect on certainty equivalent wealth for Bayesian agents.

References

Jaynes, Edwin T. 2003. *Probability theory: The logic of science*. Cambridge University Press.
