# Supplementary material for: Naked aggression: Personality and portfolio manager performance
Source: PLoS One. 2018 Feb 12;13(2):e0192630. doi: 10.1371/journal.pone.0192630 (PMC5809062; doi:10.1371/journal.pone.0192630)
Supplement: S2 File — (PDF) [file pone.0192630.s002.pdf]

## B. Instructions and Questionnaire

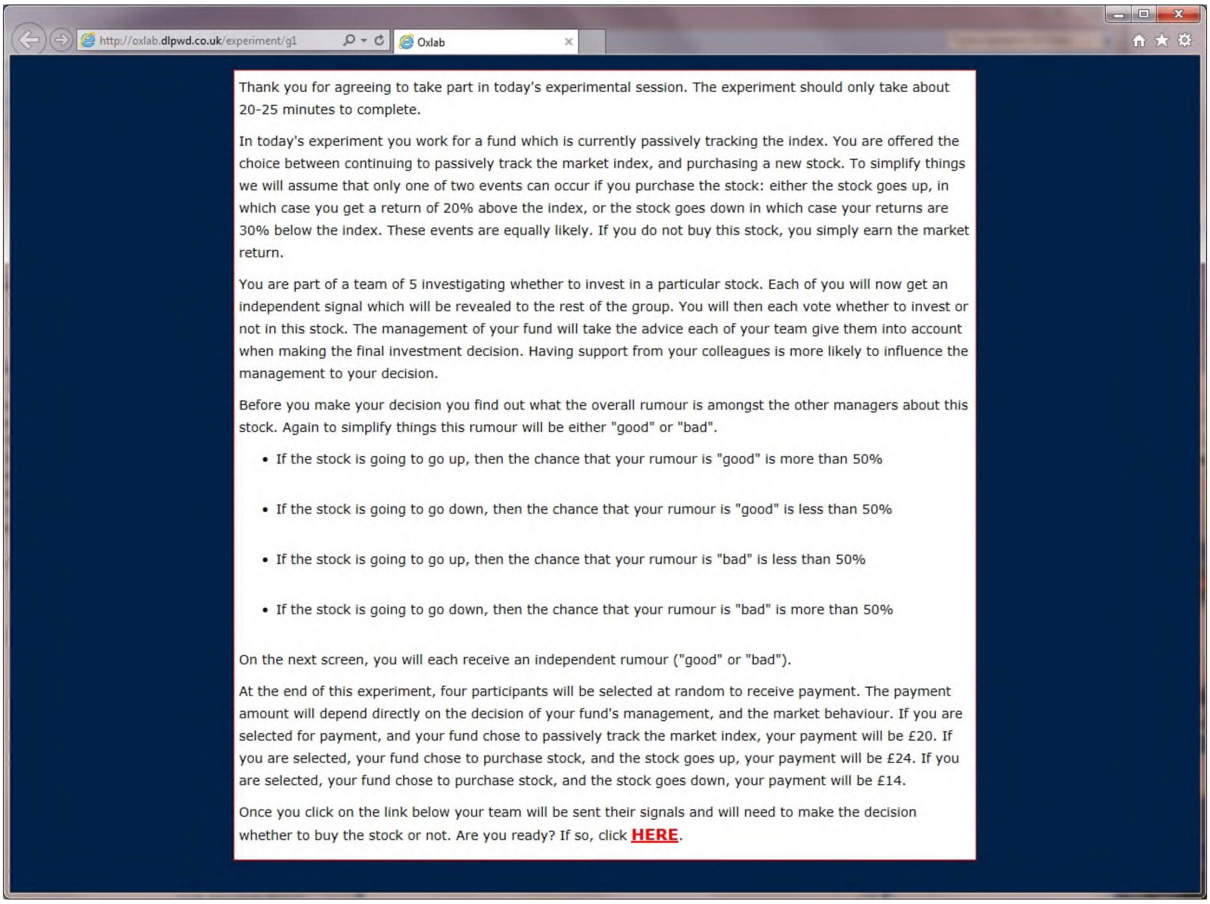

Thank you for agreeing to take part in today's experimental session. The experiment should only take about 20-25 minutes to complete.

In today's experiment you work for a fund which is currently passively tracking the index. You are offered the choice between continuing to passively track the market index, and purchasing a new stock. To simplify things we will assume that only one of two events can occur if you purchase the stock: either the stock goes up, in which case you get a return of 20% above the index, or the stock goes down in which case your returns are 30% below the index. These events are equally likely. If you do not buy this stock, you simply earn the market return.

You are part of a team of 5 investigating whether to invest in a particular stock. Each of you will now get an independent signal which will be revealed to the rest of the group. You will then each vote whether to invest or not in this stock. The management of your fund will take the advice each of your team give them into account when making the final investment decision. Having support from your colleagues is more likely to influence the management to your decision.

Before you make your decision you find out what the overall rumour is amongst the other managers about this stock. Again to simplify things this rumour will be either "good" or "bad".

- If the stock is going to go up, then the chance that your rumour is "good" is more than 50%
- If the stock is going to go down, then the chance that your rumour is "good" is less than 50%
- If the stock is going to go up, then the chance that your rumour is "bad" is less than 50%
- If the stock is going to go down, then the chance that your rumour is "bad" is more than 50%

On the next screen, you will each receive an independent rumour ("good" or "bad").

At the end of this experiment, four participants will be selected at random to receive payment. The payment amount will depend directly on the decision of your fund's management, and the market behaviour. If you are selected for payment, and your fund chose to passively track the market index, your payment will be £20. If you are selected, your fund chose to purchase stock, and the stock goes up, your payment will be £24. If you are selected, your fund chose to purchase stock, and the stock goes down, your payment will be £14.

Once you click on the link below your team will be sent their signals and will need to make the decision whether to buy the stock or not. Are you ready? If so, click **HERE**.

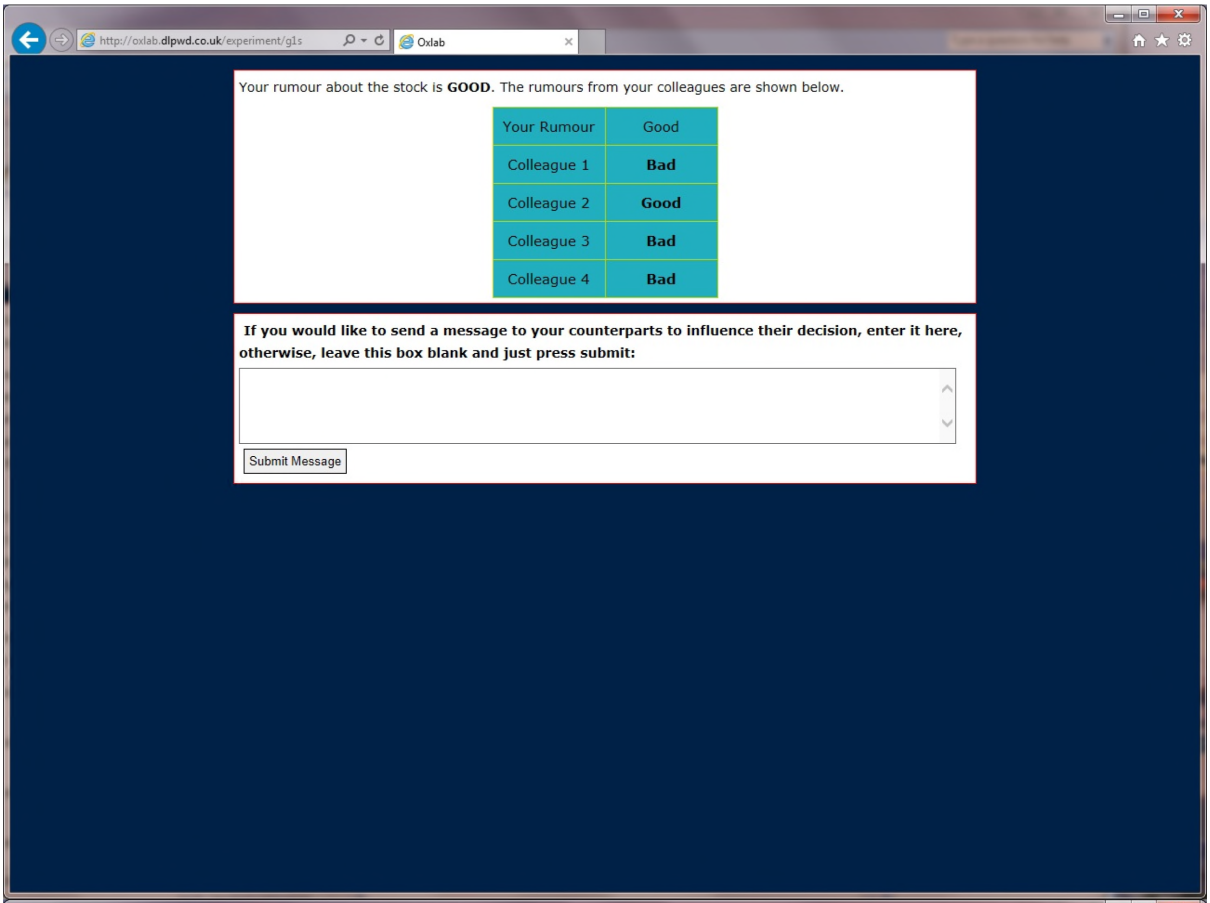
http://oxlab.dlpwd.co.uk/experiment/g1s
Oxlab
Your rumour about the stock is GOOD. The rumours from your colleagues are shown below.
Your Rumour Good
Colleague 1 Bad
Colleague 2 Good
Colleague 3 Bad
Colleague 4 Bad
If you would like to send a message to your counterparts to influence their decision, enter it here, otherwise, leave this box blank and just press submit:
Submit Message

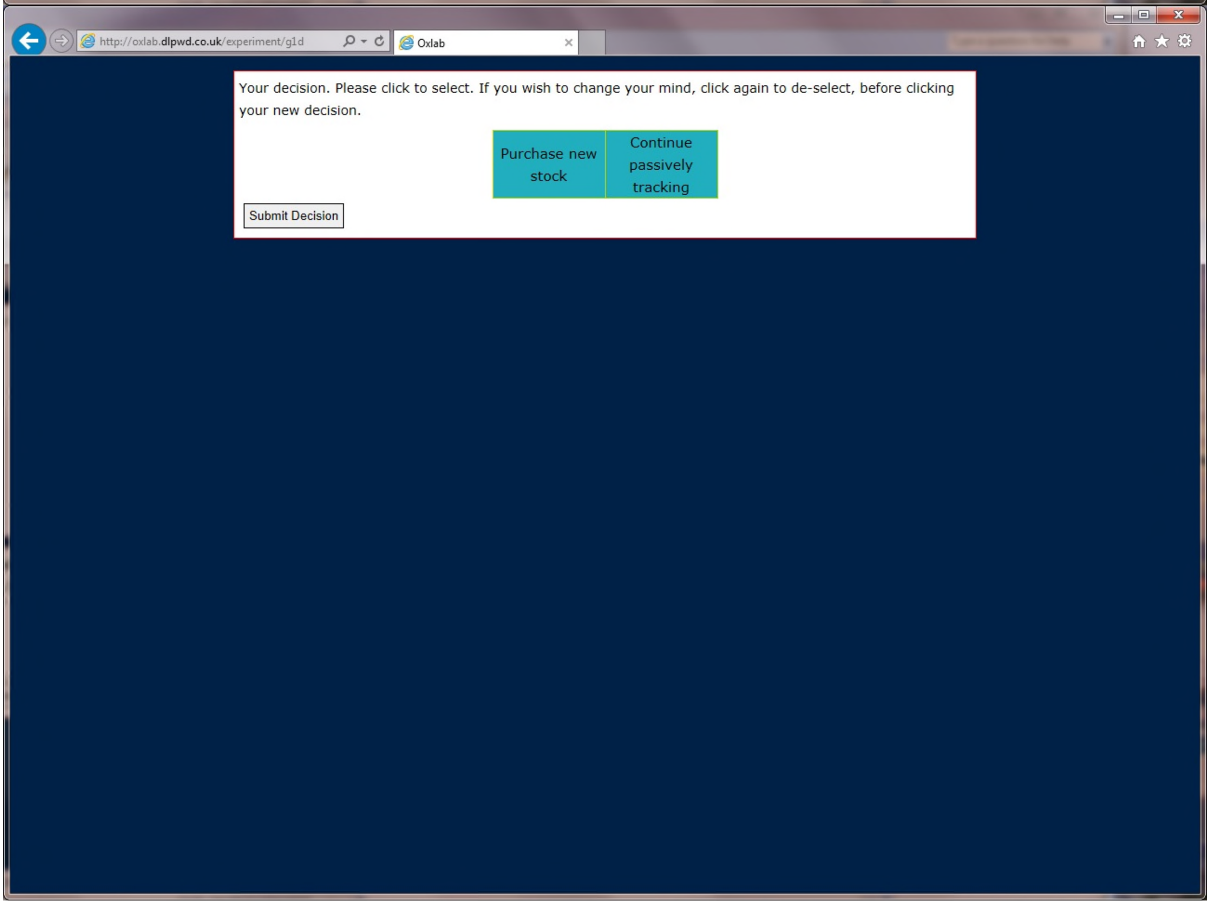
http://oxlab.dlpwd.co.uk/experiment/g1d
Oxlab
Your decision. Please click to select. If you wish to change your mind, click again to de-select, before clicking your new decision.
Purchase new stock
Continue passively tracking
Submit Decision

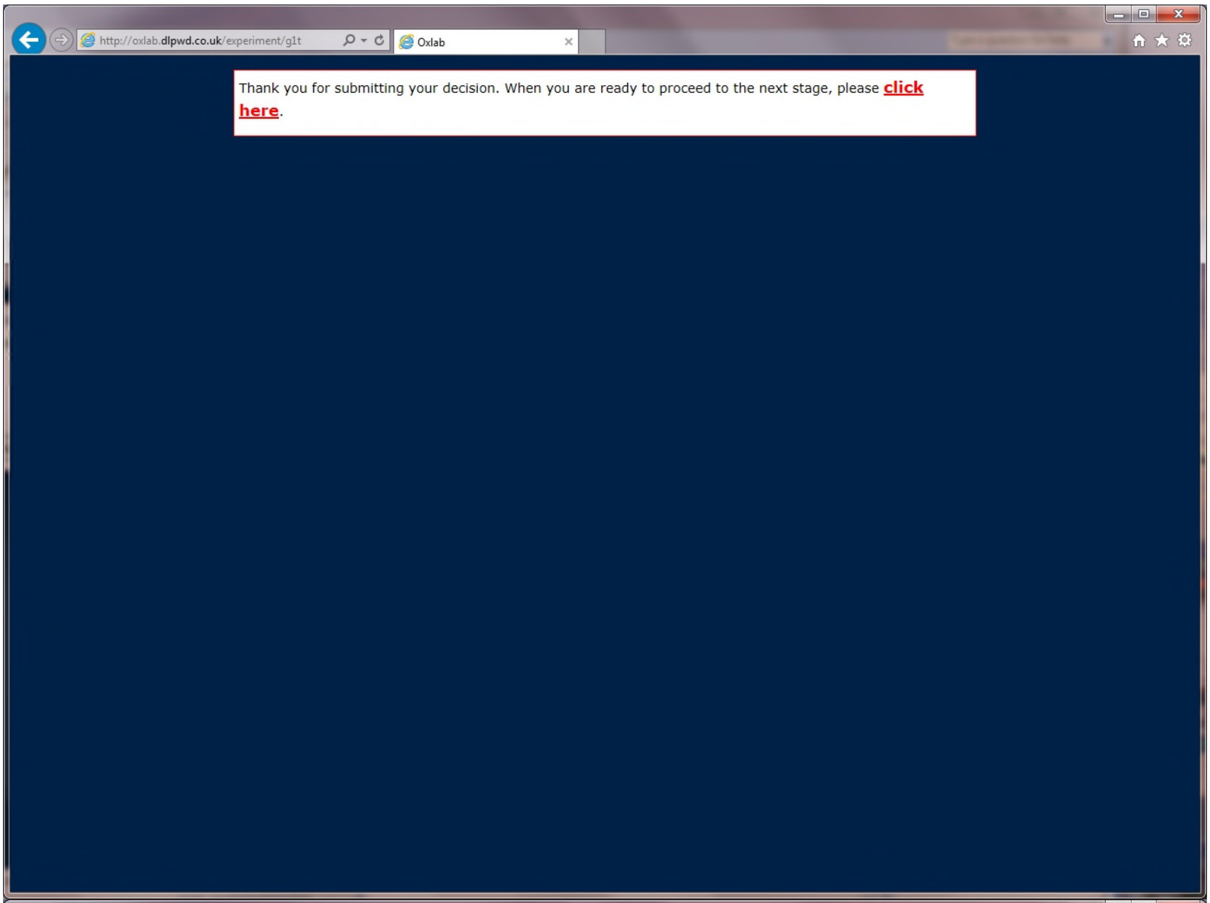
Thank you for submitting your decision. When you are ready to proceed to the next stage, please **click here**.

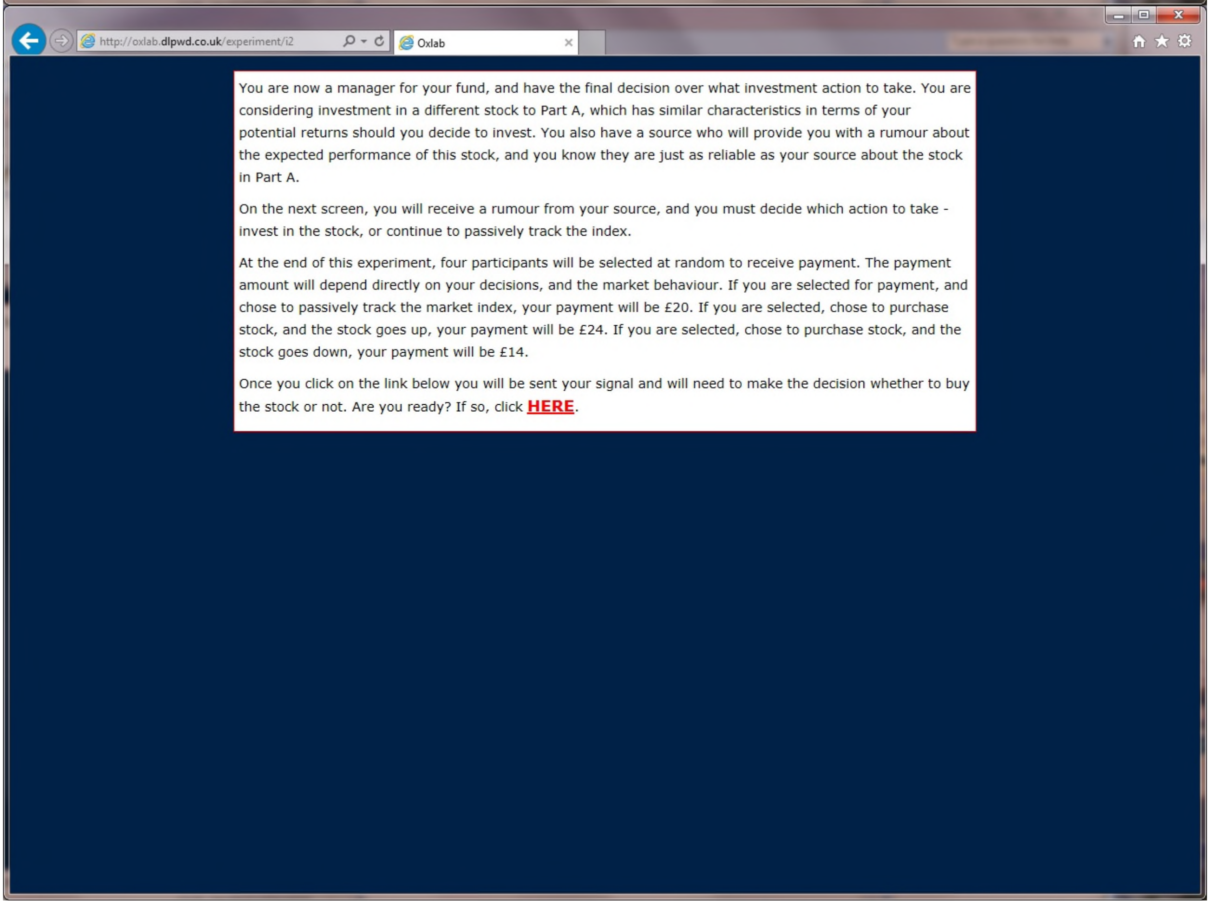
You are now a manager for your fund, and have the final decision over what investment action to take. You are considering investment in a different stock to Part A, which has similar characteristics in terms of your potential returns should you decide to invest. You also have a source who will provide you with a rumour about the expected performance of this stock, and you know they are just as reliable as your source about the stock in Part A.

On the next screen, you will receive a rumour from your source, and you must decide which action to take - invest in the stock, or continue to passively track the index.

At the end of this experiment, four participants will be selected at random to receive payment. The payment amount will depend directly on your decisions, and the market behaviour. If you are selected for payment, and chose to passively track the market index, your payment will be £20. If you are selected, chose to purchase stock, and the stock goes up, your payment will be £24. If you are selected, chose to purchase stock, and the stock goes down, your payment will be £14.

Once you click on the link below you will be sent your signal and will need to make the decision whether to buy the stock or not. Are you ready? If so, click **HERE**.

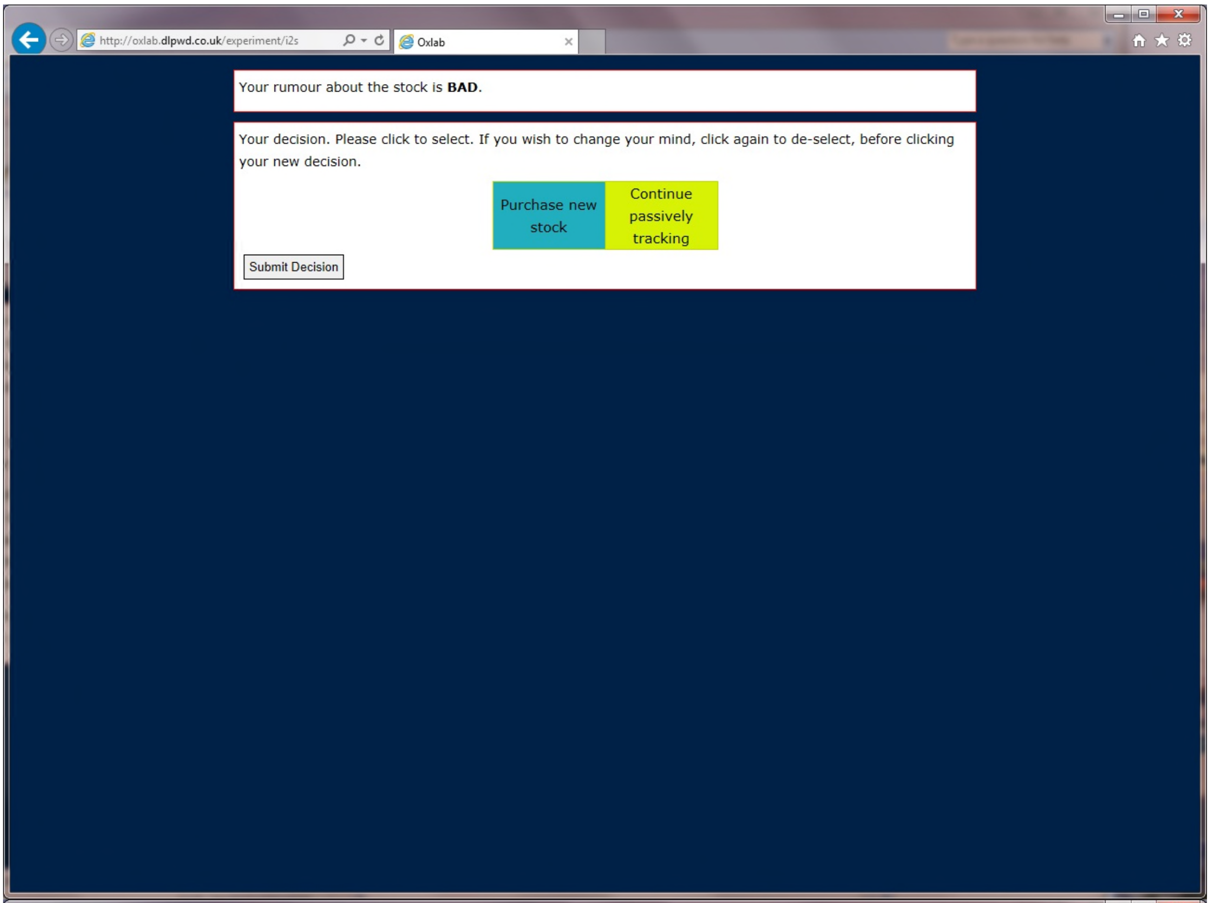
http://oxlab.dlpwd.co.uk/experiment/i2s
Oxlab
Your rumour about the stock is **BAD**.
Your decision. Please click to select. If you wish to change your mind, click again to de-select, before clicking your new decision.
Purchase new stock
Continue passively tracking
Submit Decision

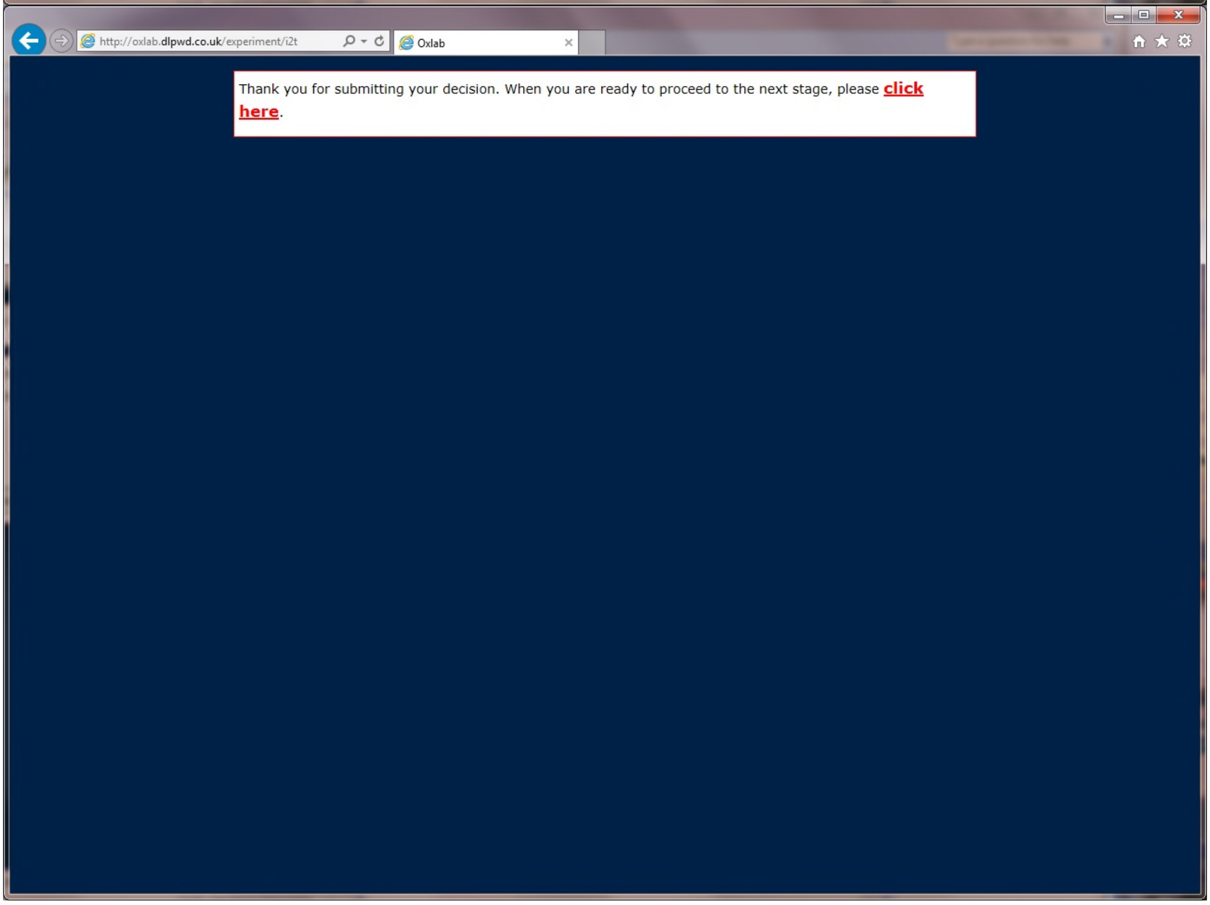
http://oxlab.dlpwd.co.uk/experiment/i2t
Oxlab
Thank you for submitting your decision. When you are ready to proceed to the next stage, please **click here**.

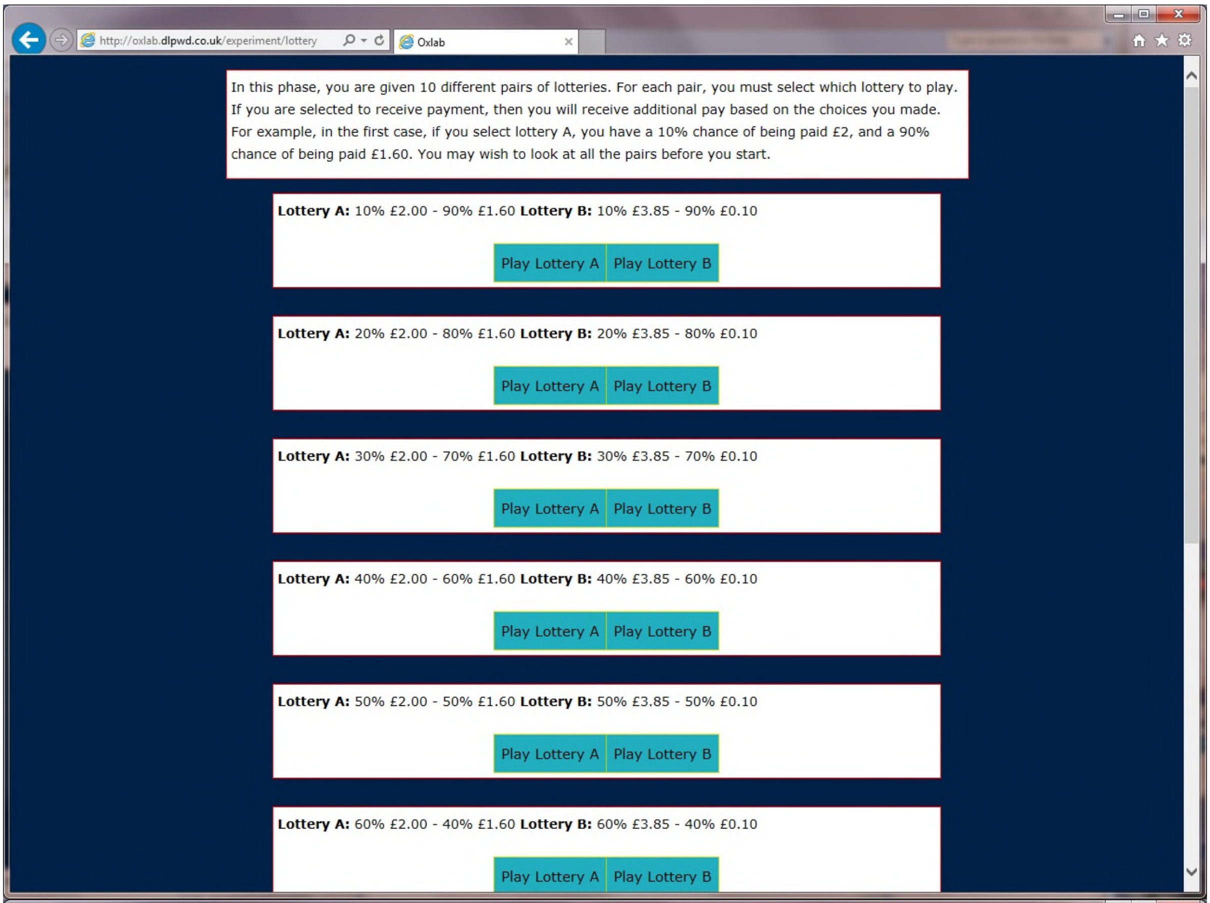
http://oxlab.dlpwd.co.uk/experiment/lottery

Oxlab

In this phase, you are given 10 different pairs of lotteries. For each pair, you must select which lottery to play. If you are selected to receive payment, then you will receive additional pay based on the choices you made. For example, in the first case, if you select lottery A, you have a 10% chance of being paid £2, and a 90% chance of being paid £1.60. You may wish to look at all the pairs before you start.

**Lottery A:** 10% £2.00 - 90% £1.60 **Lottery B:** 10% £3.85 - 90% £0.10

Play Lottery A | Play Lottery B

**Lottery A:** 20% £2.00 - 80% £1.60 **Lottery B:** 20% £3.85 - 80% £0.10

Play Lottery A | Play Lottery B

**Lottery A:** 30% £2.00 - 70% £1.60 **Lottery B:** 30% £3.85 - 70% £0.10

Play Lottery A | Play Lottery B

**Lottery A:** 40% £2.00 - 60% £1.60 **Lottery B:** 40% £3.85 - 60% £0.10

Play Lottery A | Play Lottery B

**Lottery A:** 50% £2.00 - 50% £1.60 **Lottery B:** 50% £3.85 - 50% £0.10

Play Lottery A | Play Lottery B

**Lottery A:** 60% £2.00 - 40% £1.60 **Lottery B:** 60% £3.85 - 40% £0.10

Play Lottery A | Play Lottery B

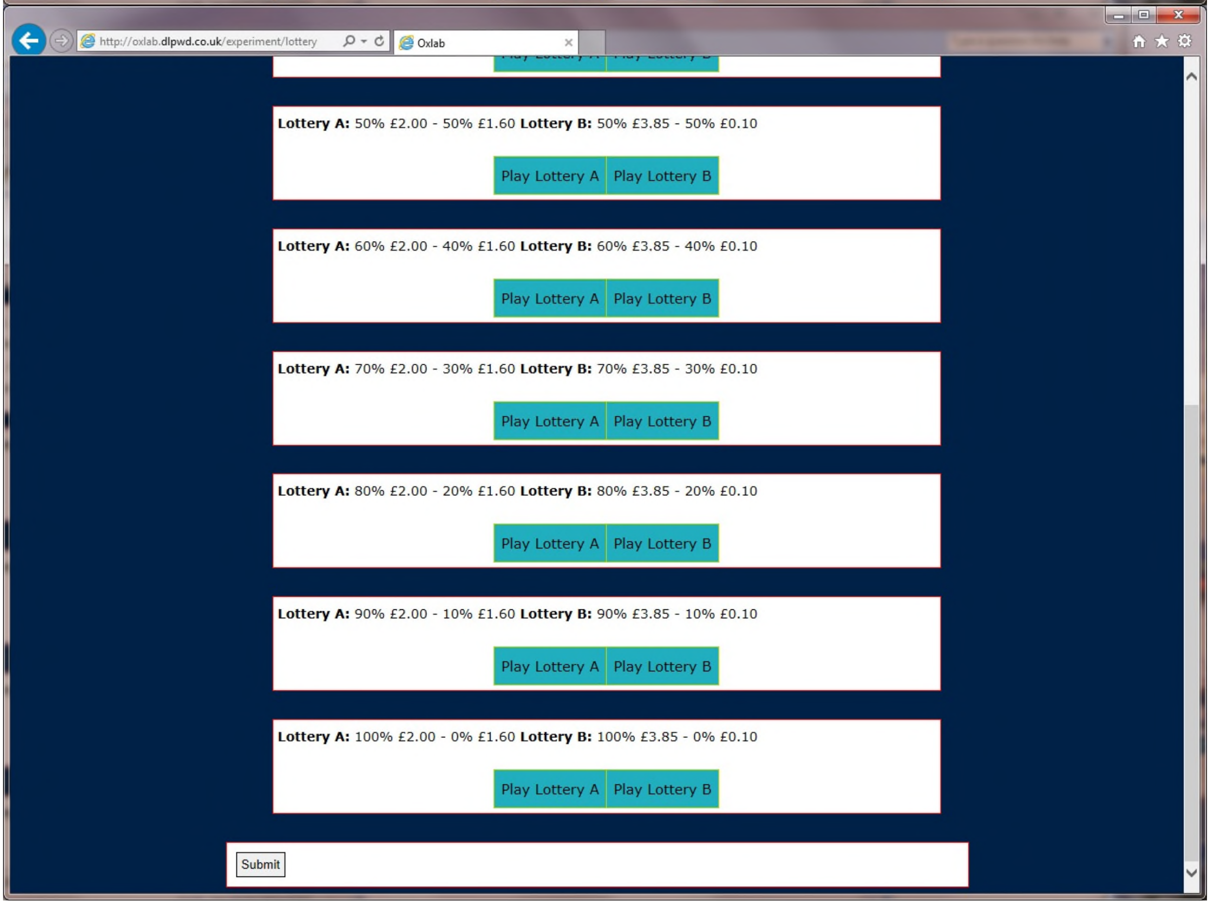
http://oxlab.dlpwd.co.uk/experiment/lottery

Oxlab

**Lottery A:** 50% £2.00 - 50% £1.60 **Lottery B:** 50% £3.85 - 50% £0.10

Play Lottery A | Play Lottery B

**Lottery A:** 60% £2.00 - 40% £1.60 **Lottery B:** 60% £3.85 - 40% £0.10

Play Lottery A | Play Lottery B

**Lottery A:** 70% £2.00 - 30% £1.60 **Lottery B:** 70% £3.85 - 30% £0.10

Play Lottery A | Play Lottery B

**Lottery A:** 80% £2.00 - 20% £1.60 **Lottery B:** 80% £3.85 - 20% £0.10

Play Lottery A | Play Lottery B

**Lottery A:** 90% £2.00 - 10% £1.60 **Lottery B:** 90% £3.85 - 10% £0.10

Play Lottery A | Play Lottery B

**Lottery A:** 100% £2.00 - 0% £1.60 **Lottery B:** 100% £3.85 - 0% £0.10

Play Lottery A | Play Lottery B

Submit

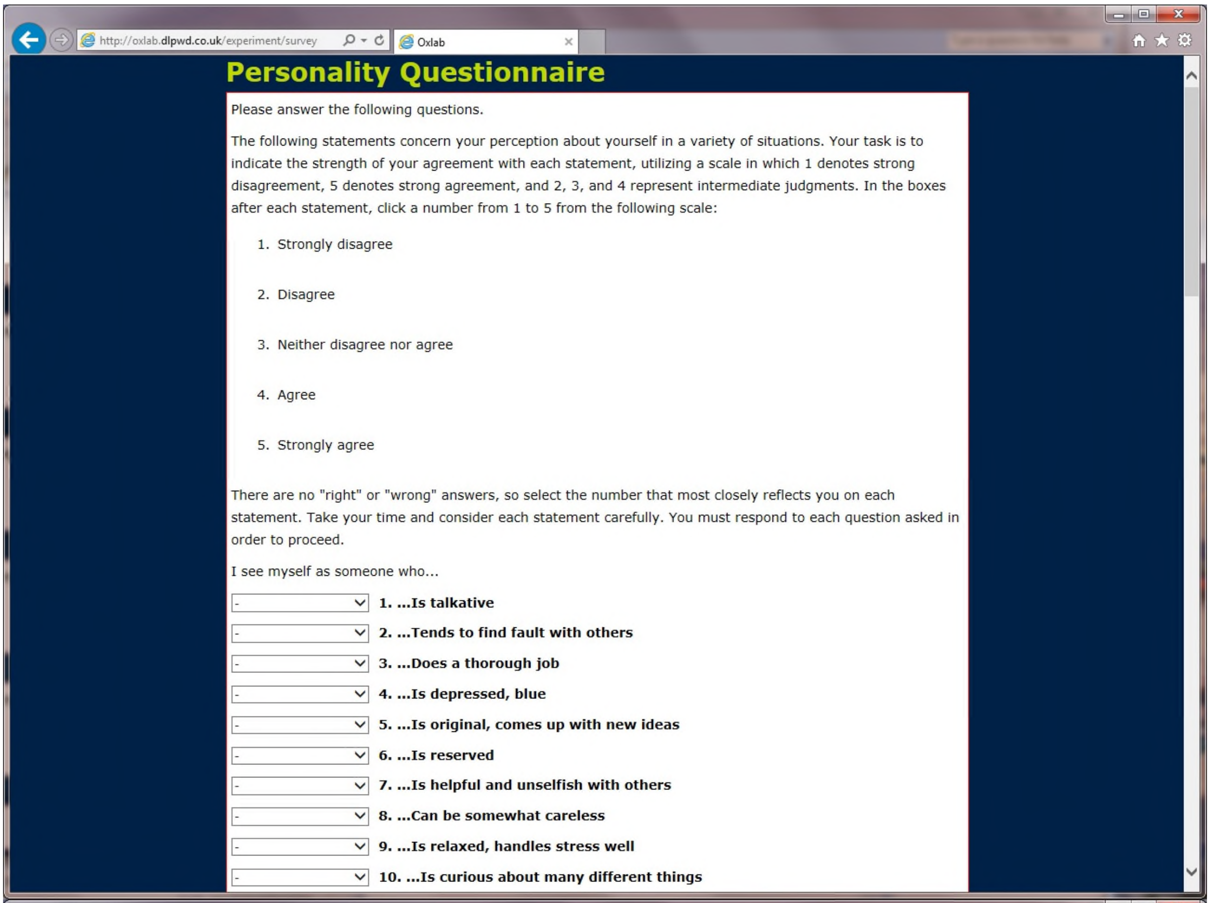

http://oxlab.dlpwd.co.uk/experiment/survey
Oxlab

# Personality Questionnaire

Please answer the following questions.

The following statements concern your perception about yourself in a variety of situations. Your task is to indicate the strength of your agreement with each statement, utilizing a scale in which 1 denotes strong disagreement, 5 denotes strong agreement, and 2, 3, and 4 represent intermediate judgments. In the boxes after each statement, click a number from 1 to 5 from the following scale:

1. Strongly disagree
2. Disagree
3. Neither disagree nor agree
4. Agree
5. Strongly agree

There are no "right" or "wrong" answers, so select the number that most closely reflects you on each statement. Take your time and consider each statement carefully. You must respond to each question asked in order to proceed.

I see myself as someone who...

- **1. ...Is talkative**
- **2. ...Tends to find fault with others**
- **3. ...Does a thorough job**
- **4. ...Is depressed, blue**
- **5. ...Is original, comes up with new ideas**
- **6. ...Is reserved**
- **7. ...Is helpful and unselfish with others**
- **8. ...Can be somewhat careless**
- **9. ...Is relaxed, handles stress well**
- **10. ...Is curious about many different things**

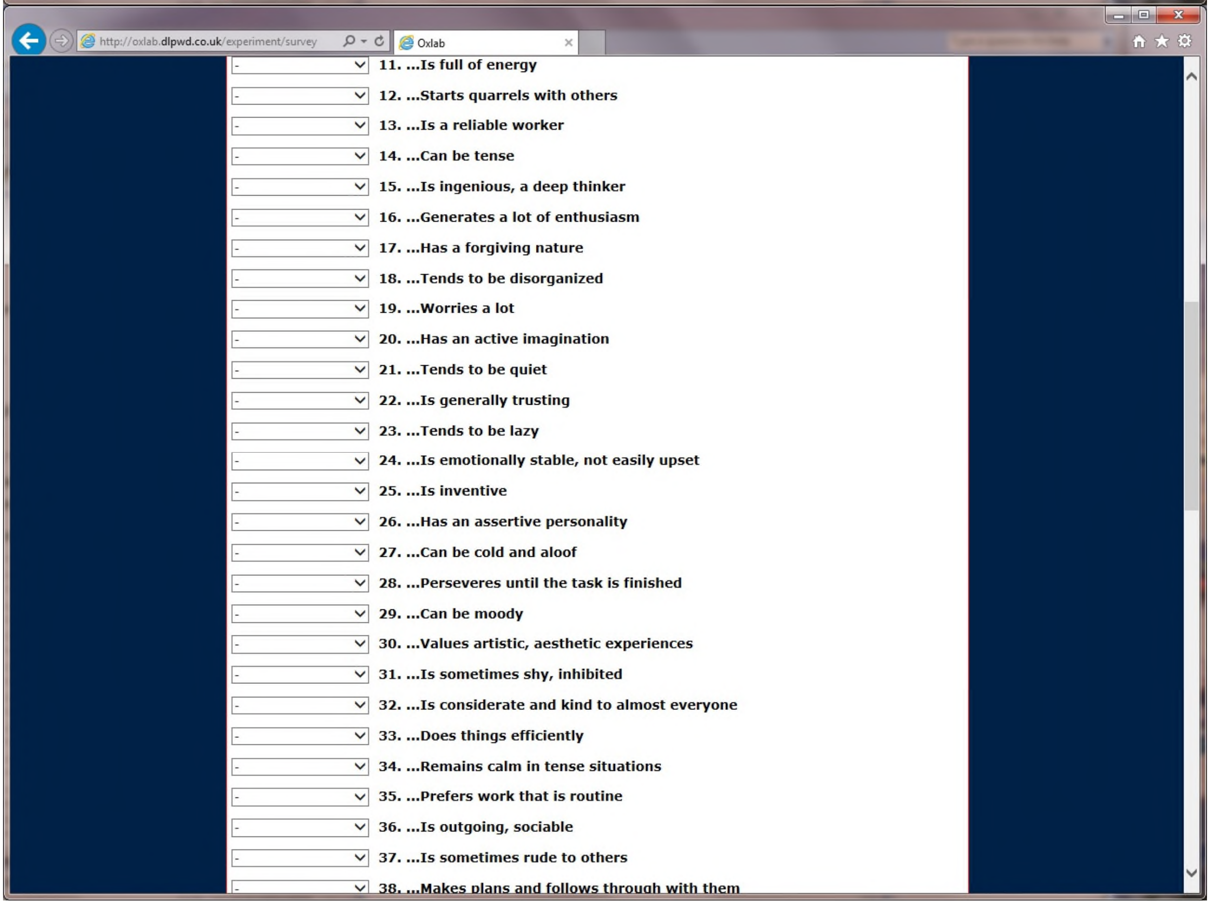

http://oxlab.dlpwd.co.uk/experiment/survey
Oxlab

- **11. ...Is full of energy**
- **12. ...Starts quarrels with others**
- **13. ...Is a reliable worker**
- **14. ...Can be tense**
- **15. ...Is ingenious, a deep thinker**
- **16. ...Generates a lot of enthusiasm**
- **17. ...Has a forgiving nature**
- **18. ...Tends to be disorganized**
- **19. ...Worries a lot**
- **20. ...Has an active imagination**
- **21. ...Tends to be quiet**
- **22. ...Is generally trusting**
- **23. ...Tends to be lazy**
- **24. ...Is emotionally stable, not easily upset**
- **25. ...Is inventive**
- **26. ...Has an assertive personality**
- **27. ...Can be cold and aloof**
- **28. ...Perseveres until the task is finished**
- **29. ...Can be moody**
- **30. ...Values artistic, aesthetic experiences**
- **31. ...Is sometimes shy, inhibited**
- **32. ...Is considerate and kind to almost everyone**
- **33. ...Does things efficiently**
- **34. ...Remains calm in tense situations**
- **35. ...Prefers work that is routine**
- **36. ...Is outgoing, sociable**
- **37. ...Is sometimes rude to others**
- **38. ...Makes plans and follows through with them**

http://oxlab.dlpwd.co.uk/experiment/survey

Oxlab

38. ...Makes plans and follows through with them

39. ...Gets nervous easily

40. ...Likes to reflect, play with ideas

41. ...Has few artistic interests

42. ...Likes to cooperate with others

43. ...Is easily distracted

44. ...Is sophisticated in art, music, or literature

45. ...Is politically liberal

For each of the following questions, please select whether you agree or disagree with the statement (please you five point scale as above)

46. I believe my success depends on ability rather than luck

47. I dislike taking responsibility for making decisions

48. I make decisions and move on

49. I believe that unfortunate evens occur because of bad luck

50. I like to take responsibility for making decisions

51. I tend to take responsibility for making decisions

52. I am always prepared

53. I leave my belongings lying around

54. I like order

55. I shirk my duties

56. I pay attention to details

57. I am exacting in my work

58. I like to follow a schedule

59. I make a mess of things

60. I often forget to put things in their proper place

61. I get my chores done right away

62. I have threatened people I know

http://oxlab.dlpwd.co.uk/experiment/survey

Oxlab

54. I like order

55. I shirk my duties

56. I pay attention to details

57. I am exacting in my work

58. I like to follow a schedule

59. I make a mess of things

60. I often forget to put things in their proper place

61. I get my chores done right away

62. I have threatened people I know

63. I sometimes feel like a powder keg ready to explode

64. Once in a while, I can't control the urge to strike another person

65. There are people that pushed me so far that we came to blows

66. I often find myself myself disagreeing with people

67. When people annoy me, I may tell them what I think of them

68. I am sometimes eaten up with jealousy

69. If I have to resort to violence to protect my rights, I will

70. Given enough provocation, I may hit another person

71. Other people always seem to get the breaks

72. I can't help getting in to arguments when other people disagree with me

73. I sometimes feel that people are laughing at me behind my back

74. How would you define yourself?

75. What do you think the 2014 percentage returns (positive or negative) will be for the Dow Jones

76. What do you think the 2014 percentage returns (positive or negative) will be for the FTSE

Submit
