# Supplementary material for: Naked aggression: Personality and portfolio manager performance
Source: PLoS One. 2018 Feb 12;13(2):e0192630. doi: 10.1371/journal.pone.0192630 (PMC5809062; doi:10.1371/journal.pone.0192630)
Supplement: S3 File — (PDF) [file pone.0192630.s003.pdf]

Questions for session

1.Thank you for agreeing to participate in this experiment. Your help with our research into financial decision making is extremely valuable and much appreciated.
This session today will take approximately 30-45 minutes. It consists of 3 different financial decision followed by a questionnaire. Please treat these decisions as if they are real, and try and decide as if this situation occurred at your work. As a bonus two of you will be picked at random, after the session, and will be paid. The exact pay will depend on the decisions you took (and what happens in the markets) but if you are picked you should expect to be paid on average £50.
When you are ready please click Next to start. Good luck!

2.In today's experiment you are a stock market analyst. You are looking at two stocks: BBCY and AACY. These two companies are in the same sector and your clients are looking to invest in only one of these two. Your job is to recommend which one.
BBCY is expected to go up or down with equal probabilities delivering to you – if you recommend it - a returns of £58 (if it went up) or £48 (if down).
AACY is different: The probabilities with which it will go up or down are initially unclear. What you know for sure is that if you recommend it and the stock goes up, then your own returns will be £68. If it goes down then your returns will be £36.

Before making your decision you received private information on AACY from a reliable source:

- If this stock is going to go up then there is over 50% chance that the information is positive
- If this stock is going to go down then there is less than 50% chance that the information is positive
- If this stock is going to go up then there is less than 50% chance that the information is negative
- If this stock is going to go down then there is over 50% chance that the information is negative

If the information is positive than there is over 50% chance that it will go up. If the information is negative then there is over 50% chance that it will go down.
Before making your decision on the next page you will be told his recommendation and more information about what other analysts in the market

are doing. Are you ready? Click Next to find out the information and make your decision.

3.Your source recommends BUYING stock AACY.
There are 10 other analysts looking at the same two stocks: 6 of them recommended BBCY, the other 4 recommend AACY.

Take a couple of minutes to think about this situation and chose one of the following:

- I recommend BBCY to my clients (46 or 58 with equal probabilities)
- I recommend to my clients to buy AACY (36 or 68, recommended by your private source)

4.In today's experiment you are a stock market analyst, part of a team of 5. Your team is looking at two stocks: BBCY and AACY. These two companies are in the same sector and your clients are looking to invest in only one of these two. Your job is to recommend which one.
BBCY is expected to go up or down with equal probabilities. If your team recommend it you can expect a returns of £58 if it went up, and £48 if it goes down.
AACY is different: The probabilities with which it will go up or down are initially unclear. What you know for sure is that if your team recommends it and the stock goes up, then you can expect returns of £68. Or if it goes down then you can expect returns of £36.

Before making your decision each of you in the group will received his/her own private information about stock AACY. Your information will come from a reliable source:

- If this stock is going to go up then there is over 50% chance that the information is positive
- If this stock is going to go down then there is less than 50% chance that the information is positive
- If this stock is going to go up then there is less than 50% chance that the information is negative
- If this stock is going to go down then there is over 50% chance that the information is negative

The other 4 members have their own independent sources, each equally well informed (although they each use different information sources and may differ in their recommendations).

Before making your decision on the next page you will be told all 5 recommendations. Are you ready? Click Next to find out the information and make your decision.

5.

The first member of the group's information suggests NOT BUYING AACY

The second member of the group's information suggests BUYING AACY

Your source recommends BUYING AACY.

The fourth member of the group's information suggests NOT BUYING AACY

The fifth and final member of the group's information suggests NOT BUYING AACY

Click here to move to the next page

6.Before making your decision you can send a message to your team mates. If you would like to do so (you do not have to send a message if you do not wish to), please enter the text in the box below. Click to move to the next page.

7.Time now to make your decision. Chose one of the following:

- I vote that our team recommends BBCY to our clients (the one with returns of 46 or 58 with equal probabilities).
- I vote that our team recommends buying AACY (the one on which the team just got private information on with possible returns of 36 or 68)

8.In today's experiment you are a stock market analyst, part of team of 4. Your team is looking at two stocks: BBCY and AACY. These two companies are in the same sector and your clients are looking to invest in only one of these two. Your job is to recommend which one.
BBCY is expected to go up or down with equal probabilities. If your team recommends it, you can expect a return of £58 if it went up, or £48 if it goes down.
AACY is different: The probabilities with which it will go up or down are initially unclear. What you know for sure is that if your team recommends it and the stock goes up, then you expect returns of £68, or if it goes down £36.

Before making your decision your team will receive private information on AACY from a reliable source:

- o If this stock is going to go up then there is over 50% chance that the information is positive

- If this stock is going to go down then there is less than 50% chance that the information is positive
- If this stock is going to go up then there is less than 50% chance that the information is negative
- If this stock is going to go down then there is over 50% chance that the information is negative

Before making your decision on the next page you will be told what your source is saying.
You will also be given information about other teams of analysts.
Are you ready? Click Next to find out the information and make your decision.

9.In total there are 5 teams looking at this sector.
3 of them recommend buying BBCY to their clients. One team recommends buying AACY.

Your source recommends BUYING stock AACY.

Take a couple of minutes to think about this situation and choose one of the following:

- I suggest our team recommend BBCY to our clients (the one with returns of 46 or 58 with equal probabilities)
- I suggest that our team recommend AACY to our clients (36 or 68, recommended by our private source).

10.Thank you for completing the financial decision part of the experiment. We now ask that you complete short risk decision task and a psychological questionnaire. There are 73 questions in total - please answer these questions truthfully. The information will be treated confidentially and according to the University normal ethical guidelines.

Click Next when you are ready.

11.On the next screen you will be making 11 choices between pairs of lotteries which we denote by Lottery A and Lottery B.

Lottery A will always give you either £2 or £1.60 while Lottery B will always give you either £3.85 or £0.1

Below we list the 11 choices, please take a look at these before coming back to reading this text.

In the first choice (choice 1), Lottery A is clearly better: You get £1.60 for sure as

opposed to B which gives you £0.1 for sure.

Similarly, Lottery B is better in the last choice (choice 11) because you get £3.85 for sure, whereas if you go with A you will only get £2.

As the probabilities change you might want to switch from lottery A to B. Take a look at the list below and tell us at what stage you would like to switch

So, for example if you say choice 2, that means that you will prefer lottery B to A in choices 2-11. If you say choice 11 this means you prefer A in 1-10 and only like B at the last choice.

Please read carefully and take your time before making your choice:

12.Choice 1: Lottery A: 0% £2.00 - 100% £1.60 Lottery B: 0% £3.85 - 100% £0.10

Choice 2: Lottery A: 10% £2.00 - 90% £1.60 Lottery B: 10% £3.85 - 90% £0.10

Choice 3: Lottery A: 20% £2.00 - 80% £1.60 Lottery B: 20% £3.85 - 80% £0.10

Choice 4: Lottery A: 30% £2.00 - 70% £1.60 Lottery B: 30% £3.85 - 70% £0.10

Choice 5: Lottery A: 40% £2.00 - 60% £1.60 Lottery B: 40% £3.85 - 60% £0.10

Choice 6: Lottery A: 50% £2.00 - 50% £1.60 Lottery B: 50% £3.85 - 50% £0.10

Choice 7: Lottery A: 60% £2.00 - 40% £1.60 Lottery B: 60% £3.85 - 40% £0.10

Choice 8: Lottery A: 70% £2.00 - 30% £1.60 Lottery B: 70% £3.85 - 30% £0.10

Choice 9: Lottery A: 80% £2.00 - 20% £1.60 Lottery B: 80% £3.85 - 20% £0.10

Choice 10: Lottery A: 90% £2.00 - 10% £1.60 Lottery B: 90% £3.85 - 10% £0.10

Choice 11: Lottery A: 100% £2.00 - 0% £1.60 Lottery B: 100% £3.85 - 0% £0.10

- 1
- 2
- 3
- 4
- 5
- 6

- 7
- 8
- 9
- 10
- 11

13.What is your Gender?

- Female
- Male

14.What is your age?

15.How many years of experience you have in finance, and/or of making finance related decisions at your work place (please send zero if you do not have any experience in finance)?

16.The following statements concern your perception about yourself in a variety of situations. Your task is to indicate the strength of your agreement with each statement, utilizing a scale in which 1 denotes strong disagreement, 5 denotes strong agreement, and 2, 3, and 4 represent intermediate judgments. In the boxes after each statement, click a number from 1 to 5 from the following scale:
1.Strongly disagree
2.Disagree
3.Neither disagree nor agree
4.Agree
5.Strongly agree
There are no "right" or "wrong" answers, so select the number that most closely reflects you on each statement. Take your time and consider each statement carefully. You must respond to each question asked in order to proceed.
I see myself as someone who...

...Is talkative

- Strongly disagree
- Disagree
- Neither disagree nor agree
- Agree
- Strongly agree

17....Tends to find fault with others

- Strongly disagree
- Disagree
- Neither disagree nor agree

- Agree
- Strongly agree

18....Does a thorough job

- Strongly disagree
- Disagree
- Neither disagree nor agree
- Agree
- Strongly agree

19....Is depressed, blue

- Strongly disagree
- Disagree
- Neither disagree nor agree
- Agree
- Strongly agree

20....Is original, comes up with new ideas

- Strongly disagree
- Disagree
- Neither disagree nor agree
- Agree
- Strongly agree

21....Is reserved

- Strongly disagree
- Disagree
- Neither disagree nor agree
- Agree
- Strongly agree

22....Is helpful and unselfish with others

- Strongly disagree
- Disagree
- Neither disagree nor agree
- Agree
- Strongly agree

23....Can be somewhat careless

- Strongly disagree
- Disagree
- Neither disagree nor agree
- Agree
- Strongly agree

24....Is relaxed, handles stress well

- Strongly disagree
- Disagree
- Neither disagree nor agree
- Agree
- Strongly agree

25....Is curious about many different things

- Strongly disagree
- Disagree
- Neither disagree nor agree
- Agree
- Strongly agree

26....Is full of energy

- Strongly disagree
- Disagree
- Neither disagree nor agree
- Agree
- Strongly agree

27....Starts quarrels with others

- Strongly disagree
- Disagree
- Neither disagree nor agree
- Agree
- Strongly agree

28....Is a reliable worker

- Strongly disagree

- Disagree
- Neither disagree nor agree
- Agree
- Strongly agree

29....Can be tense

- Strongly disagree
- Disagree
- Neither disagree nor agree
- Agree
- Strongly agree

30....Is ingenious, a deep thinker

- Strongly disagree
- Disagree
- Neither disagree nor agree
- Agree
- Strongly agree

31....Generates a lot of enthusiasm

- Strongly disagree
- Disagree
- Neither disagree nor agree
- Agree
- Strongly agree

32....Has a forgiving nature

- Strongly disagree
- Disagree
- Neither disagree nor agree
- Agree
- Strongly agree

33....Tends to be disorganized

- Strongly disagree
- Disagree
- Neither disagree nor agree
- Agree

- Strongly agree

34....Worries a lot

- Strongly disagree
- Disagree
- Neither disagree nor agree
- Agree
- Strongly agree

35....Has an active imagination

- Strongly disagree
- Disagree
- Neither disagree nor agree
- Agree
- Strongly agree

36....Tends to be quiet

- Strongly disagree
- Disagree
- Neither disagree nor agree
- Agree
- Strongly agree

37....Is generally trusting

- Strongly disagree
- Disagree
- Neither disagree nor agree
- Agree
- Strongly agree

38....Tends to be lazy

- Strongly disagree
- Disagree
- Neither disagree nor agree
- Agree
- Strongly agree

39....Is emotionally stable, not easily upset

- Strongly disagree
- Disagree
- Neither disagree nor agree
- Agree
- Strongly agree

40....Is inventive

- Strongly disagree
- Disagree
- Neither disagree nor agree
- Agree
- Strongly agree

41....Has an assertive personality

- Strongly disagree
- Disagree
- Neither disagree nor agree
- Agree
- Strongly agree

42....Can be cold and aloof

- Strongly disagree
- Disagree
- Neither disagree nor agree
- Agree
- Strongly agree

43....Perseveres until the task is finished

- Strongly disagree
- Disagree
- Neither disagree nor agree
- Agree
- Strongly agree

44....Can be moody

- Strongly disagree
- Disagree
- Neither disagree nor agree

- Agree
- Strongly agree

45....Values artistic, aesthetic experiences

- Strongly disagree
- Disagree
- Neither disagree nor agree
- Agree
- Strongly agree

46....Is sometimes shy, inhibited

- Strongly disagree
- Disagree
- Neither disagree nor agree
- Agree
- Strongly agree

47....Is considerate and kind to almost everyone

- Strongly disagree
- Disagree
- Neither disagree nor agree
- Agree
- Strongly agree

48....Does things efficiently

- Strongly disagree
- Disagree
- Neither disagree nor agree
- Agree
- Strongly agree

49....Remains calm in tense situations

- Strongly disagree
- Disagree
- Neither disagree nor agree
- Agree
- Strongly agree

50....Prefers work that is routine

- Strongly disagree
- Disagree
- Neither disagree nor agree
- Agree
- Strongly agree

51....Is outgoing, sociable

- Strongly disagree
- Disagree
- Neither disagree nor agree
- Agree
- Strongly agree

52....Is sometimes rude to others

- Strongly disagree
- Disagree
- Neither disagree nor agree
- Agree
- Strongly agree

53....Makes plans and follows through with them

- Strongly disagree
- Disagree
- Neither disagree nor agree
- Agree
- Strongly agree

54....Gets nervous easily

- Strongly disagree
- Disagree
- Neither disagree nor agree
- Agree
- Strongly agree

55....Likes to reflect, play with ideas

- Strongly disagree

- Disagree
- Neither disagree nor agree
- Agree
- Strongly agree

56....Has few artistic interests

- Strongly disagree
- Disagree
- Neither disagree nor agree
- Agree
- Strongly agree

57....Likes to cooperate with others

- Strongly disagree
- Disagree
- Neither disagree nor agree
- Agree
- Strongly agree

58....Is easily distracted

- Strongly disagree
- Disagree
- Neither disagree nor agree
- Agree
- Strongly agree

59....Is sophisticated in art, music, or literature

- Strongly disagree
- Disagree
- Neither disagree nor agree
- Agree
- Strongly agree

60....Is politically liberal

- Strongly disagree
- Disagree
- Neither disagree nor agree
- Agree

- Strongly agree

61. For each of the following questions, please select whether you agree or disagree with the statement (please you five point scale as above)
I believe my success depends on ability rather than luck

- Strongly disagree
- Disagree
- Neither disagree nor agree
- Agree
- Strongly agree

62. I dislike taking responsibility for making decisions

- Strongly disagree
- Disagree
- Neither disagree nor agree
- Agree
- Strongly agree

63. I make decisions and move on

- Strongly disagree
- Disagree
- Neither disagree nor agree
- Agree
- Strongly agree

64. I believe that unfortunate events occur because of bad luck

- Strongly disagree
- Disagree
- Neither disagree nor agree
- Agree
- Strongly agree

65. I like to take responsibility for making decisions

- Strongly disagree
- Disagree
- Neither disagree nor agree
- Agree
- Strongly agree

66.I tend to take responsibility for making decisions

- Strongly disagree
- Disagree
- Neither disagree nor agree
- Agree
- Strongly agree

67.I am always prepared

- Strongly disagree
- Disagree
- Neither disagree nor agree
- Agree
- Strongly agree

68.I leave my belongings lying around

- Strongly disagree
- Disagree
- Neither disagree nor agree
- Agree
- Strongly agree

69.I like order

- Strongly disagree
- Disagree
- Neither disagree nor agree
- Agree
- Strongly agree

70.I shirk my duties

- Strongly disagree
- Disagree
- Neither disagree nor agree
- Agree
- Strongly agree

71.I pay attention to details

- Strongly disagree

- Disagree
- Neither disagree nor agree
- Agree
- Strongly agree

72.I am exacting in my work

- Strongly disagree
- Disagree
- Neither disagree nor agree
- Agree
- Strongly agree

73.I like to follow a scq76le

- Strongly disagree
- Disagree
- Neither disagree nor agree
- Agree
- Strongly agree

74.I make a mess of things

- Strongly disagree
- Disagree
- Neither disagree nor agree
- Agree
- Strongly agree

75.I often forget to put things in their proper place

- Strongly disagree
- Disagree
- Neither disagree nor agree
- Agree
- Strongly agree

76.I get my chores done right away

- Strongly disagree
- Disagree
- Neither disagree nor agree
- Agree

- Strongly agree

77.I have threatened people I know

- Strongly disagree
- Disagree
- Neither disagree nor agree
- Agree
- Strongly agree

78.I sometimes feel like a powder keg ready to explode

- Strongly disagree
- Disagree
- Neither disagree nor agree
- Agree
- Strongly agree

79.Once in a while, I can't control the urge to strike another person

- Strongly disagree
- Disagree
- Neither disagree nor agree
- Agree
- Strongly agree

80.There are people that pushed me so far that we came to blows

- Strongly disagree
- Disagree
- Neither disagree nor agree
- Agree
- Strongly agree

81.I often find myself myself disagreeing with people

- Strongly disagree
- Disagree
- Neither disagree nor agree
- Agree
- Strongly agree

82.When people annoy me, I may tell them what I think of them

- Strongly disagree
- Disagree
- Neither disagree nor agree
- Agree
- Strongly agree

83.I am sometimes eaten up with jealousy

- Strongly disagree
- Disagree
- Neither disagree nor agree
- Agree
- Strongly agree

84.If I have to resort to violence to protect my rights, I will

- Strongly disagree
- Disagree
- Neither disagree nor agree
- Agree
- Strongly agree

85.Given enough provocation, I may hit another person

- Strongly disagree
- Disagree
- Neither disagree nor agree
- Agree
- Strongly agree

86.Other people always seem to get the breaks

- Strongly disagree
- Disagree
- Neither disagree nor agree
- Agree
- Strongly agree

87.I can't help getting in to arguments when other people disagree with me

- Strongly disagree
- Disagree
- Neither disagree nor agree

- Agree
- Strongly agree

88. I sometimes feel that people are laughing at me behind my back

- Strongly disagree
- Disagree
- Neither disagree nor agree
- Agree
- Strongly agree
