# Supplementary material for: Naked aggression: Personality and portfolio manager performance
Source: PLoS One. 2018 Feb 12;13(2):e0192630. doi: 10.1371/journal.pone.0192630 (PMC5809062; doi:10.1371/journal.pone.0192630)
Supplement: S4 File — (PDF) [file pone.0192630.s004.pdf]

id	Individual D	Group Deci	Risk	Gender	Age	Experience	Extraversio	Agreeabler
9	0	0	7	1	30-40	2.5	18	32
23	1	0	5	1	30-40	7	32	33
24	1	0	4	0	20-30	0.5	28	39
39	1	0	6	1	20-30	4	29	29
55	0	0	7	1	20-30	4	27	37
56	0	0	5	1	20-30	3	24	30
66	0	0	3	1	30-40	4	35	43
77	0	0	7	1	30-40	9	33	27
82	0		5	1	30-40	8	30	36
83	0	0	5	1	30-40	9	32	40
85	1		3	1	30-40	8	32	39
87	0	1	5	1	30-40	9	34	28
200	1	0	5	1	30-40	12	21	34
201	0	0	1	0	30-40	0	25	39
202	0	1	5	1	30-40	15	21	24
204	0	1	10	0	40-50	8	33	34
205	0	0	6	1	40-50	26	25	29
206	0	0	4	1	40-50	9	29	34
207	1	1	4	1	40-50	15	24	31
208	0	0	7	1	20-30	8	28	33
209	0	0	6	1	40-50	18	32	36
211	0	0	5	0	30-40	8	19	39
212	1	0	NO GOOD	1	20-30	5	28	37
213	0	0	9	0	20-30	9	39	30
214	0	1	7	1	20-30	7	31	27
215	0	0	8	1	20-30	8	27	32
216	1	0	5	m	50-60	28	21	33
217	0	0	5	1	20-30	7	37	32
218	1	1	5	1	20-30	8	30	33
220	1	0	5	0	50-60	27	29	33
221	1	0	6	1	40-50	12	25	20
222	0	1	6	1	20-30	9	17	37
223	0	1	NO GOOD	1	30-40	7	27	33
224	0	0	5	1	40-50	25	29	32
225	0	0	4	1	40-50	11	24	38
226	0	0	7	1	20-30	5	33	35
227	1	1	3	1	40-50	9	26	31
228	0	0	3	1	40-50	23	29	38
230	1	1	4	1	40-50	24	25	35
231	0	0	5	1	50-60	18	28	30
232	0	0	5	1	20-30	5	23	33
233	0	0	NO GOOD	1	20-30	21	26	37
234	0	0	4	0	20-30	14	28	31
235	1	0	5	0	20-30	8	37	41
236	0	0	4	F	20-30	12	36	35
237	0	1	NO GOOD	M	20-30	15	32	32
238	0	0	6	1	40-50	8	27	29
239	0	0	7	1	30-40	11	35	38
240	0	0	7	1	50-60	25	29	27

241	1	1	4	1	20-30		1	30	37
242	0	0	1	1	20-30		8	33	36
243	1	1	8	0	40-50	10+		32	39

Conscientic	Neuroticisr	Openness	Rotter	Aggressiveness
26	26	38	2	29
35	20	44	2	25
38	18	35	2	17
45	18	39	4	46
27	16	36	1	25
40	22	38	3	24
36	11	34	4	18
37	10	35	4	27
32	16	40	4	31
32	18	41	3	21
39	27	42	3	22
30	19	38	2	34
35	20	33	2	31
31	24	50	3	15
27	28	37	2	35
44	13	42	4	30
34	28	45	2	25
33	20	36	3	18
30	17	39	3	30
35	24	38	3	30
33	22	40	3	27
36	16	29	3	14
40	19	38	4	27
35	15	40	2	21
40	20	39	4	44
41	18	39	1	30
31	17	37	2	27
32	31	35	3	41
38	23	39	4	18
39	31	42	3	19
29	22	39	2	31
35	28	32	0	43
38	14	31	4	16
33	22	38	4	21
26	30	43	-2	26
33	13	31	3	18
25	29	45	2	35
44	16	36	3	18
36	20	33	2	26
36	18	45	2	21
31	22	33	4	36
39	16	33	2	16
36	23	39	2	34
42	16	46	4	16
37	27	40	4	16
40	14	36	6	34
39	33	33	2	23
40	14	40	3	23
44	25	41	3	24

31	23	32	3	28
28	14	34	3	25
30	17	47	2	13
