# Supplementary material for: Naked aggression: Personality and portfolio manager performance
Source: PLoS One. 2018 Feb 12;13(2):e0192630. doi: 10.1371/journal.pone.0192630 (PMC5809062; doi:10.1371/journal.pone.0192630)
Supplement: S5 File — (PDF) [file pone.0192630.s005.pdf]

ID	D1	D2	D3	HL	Gender	Age	Exp	Extra
1	1	1	1	6	Male	40	15	28
2	1	1	1	3	Female	37	17	33
3	0	1	1	11	Female	39	10	33
4	0	0	0	6	Female	30	3	27
5	1	0	1	11	Male	34	8	33
6	1	0	0	1	Female	48	10	23
7	1	0	1	2	Male	40	15	34
8	1	0	1	7	Male	29	2	34
9	0	1	0	7	Female	35	10	31
10	0	0	1	11	Female	33	1	26
11	1	1	1	11	Female	47	10	31
12	1	1	1	8	Male	31	10	23
13	1	1	1	1	Male	36	10	28
14	0	0	1	8	Male	48	6	24
15	1	0	1	6	Male	32	1	22
16	0	0	0	8	Male	31	1	27
17	1	1	0	8	Male	32	1	33
18	1	0	0	11	Male	42	20	18
19	1	1	1	9	Male	31	8	33
20	0	0	0	5	Male	38	13	19
21	1	0	1	1	Female	25	5	31
22	0	0	0	5	Male	35	1	19
23	0	1	0	1	Male	28	3	27
24	0	0	0	11	Male	48	10	35
25	0	1	1	6	Female	35	5	27
26	0	1	0	6	Female	34	6	28
27	0	0	0	6	Female	26	5	32
28	0	1	1	7	Female	32	1	33
29	0	0	0	3	Female	30	8	22
30	0	0	0	4	Female	40	10	33
31	0	0	1	1	Male	39	10	26
32	0	0	0	1	Male	44	15	28
33	1	0	1	7	Male	43	8	32
34	1	0	0	6	Male	46	20	23
35	0	0	1	7	Male	42	23	40
36	1	1	1	11	Male	33	4	33
37	1	0	1	11	Male	57	20	27
38	0	1	0	1	Male	31	10	28
39	1	1	1	11	Male	40	19	25
40	0	0	0	7	Male	33	8	28
41	0	0	0	6	Female	40	16	28
42	0	1	0	1	Male	44	15	32
43	0	1	0	6	Male	50	23	26
44	0	1	0	11	Female	33	8	29
45	0	0	0	6	Male	31	8	28
46	1	1	0	8	Male	41	12	23
47	0	1	0	11	Male	38	8	28
48	1	0	1	4	Male	34	9	24
49	0	0	0	11	Male	34	10	32

50	0	0	0	6	Male	36	8	28
51	0	1	1	6	Male	33	5	26
52	0	1	0	9	Male	29	5	27
53	1	0	1	6	Female	34	5	39
54	0	0	0	11	Male	47	0	29
55	0	1	1	6	Female	32	10	27
56	0	0	0	11	Male	42	8	26
57	1	0	1	11	Male	50	24	32
58	0	0	0	4	Male	32	7	30
59	1	1	1	6	Male	45	20	34
60	0	0	0	5	Male	52	8	17
61	0	0	0	6	Male	27	4	28
62	1	1	1	11	Male	37	5	34
63	0	0	0	9	Male	31	4	30
64	0	1	0	11	Male	30	9	32
65	1	1	0	6	Male	38	2	25
66	1	1	1	11	Male	52	30	32
67	0	0	0	11	Male	52	26	39
68	0	0	0	10	Male	36	3	26
69	0	0	0	4	Male	27	0.3	28
70	1	1	1	4	Female	32	3	33
71	1	0	1	2	Male	38	10	22
72	0	0	0	8	Female	36	10	27

Agree	Consc	Neuro	Open	Richter	Agg
39	42	16	40	25	29
25	32	25	35	20	39
37	38	20	37	20	30
38	37	16	31	25	14
38	29	18	37	22	20
39	37	24	42	26	21
42	44	14	42	41	23
40	38	13	41	34	19
31	38	24	37	23	29
24	38	33	41	13	25
35	32	30	42	15	38
23	28	26	42	24	30
33	26	24	37	25	49
38	36	21	40	31	17
25	33	20	27	30	20
27	33	23	31	20	23
31	31	27	40	28	36
33	42	28	31	29	23
28	34	26	38	30	35
35	35	15	35	25	25
33	33	21	44	22	24
29	26	23	36	13	24
31	33	29	35	20	31
32	34	17	38	17	26
37	35	22	33	25	28
25	36	23	40	31	34
37	37	15	37	37	15
24	37	27	37	24	41
25	31	24	36	17	25
36	31	19	36	14	23
42	45	22	41	39	16
38	40	14	35	37	16
34	35	19	39	27	24
33	34	17	35	29	23
35	24	28	40	9	20
29	36	16	42	21	36
33	37	22	41	39	22
25	39	18	36	27	32
36	34	21	38	29	34
31	34	23	40	23	28
38	39	14	45	37	28
30	37	21	40	32	32
38	31	18	33	17	34
33	37	15	37	21	36
34	30	18	39	21	26
27	40	33	38	32	32
36	29	16	37	18	36
34	30	25	40	20	32
34	34	15	37	20	27

39	43	15	38	31	21
18	31	24	36	22	44
30	30	22	36	20	38
26	21	22	40	18	35
34	31	13	45	24	33
26	36	28	43	34	36
32	31	28	30	16	26
37	31	21	40	19	20
34	31	28	29	20	26
40	32	10	44	33	17
29	29	30	31	18	22
40	33	17	39	28	18
33	36	21	48	22	25
31	34	19	37	23	17
38	38	17	36	25	27
34	38	13	44	25	17
45	43	16	46	37	31
22	37	25	40	28	17
32	43	16	32	34	31
34	31	29	39	22	25
25	32	17	47	20	32
32	37	23	36	26	26
35	41	14	40	32	20
